# Helminth driven gut inflammation and microbial translocation associate with altered vaccine responses in rural Uganda

**DOI:** 10.1038/s41541-025-01116-x

**Published:** 2025-03-26

**Authors:** Jacent Nassuuna, Joas Sterk, Bridgious Walusimbi, Agnes Natukunda, Ronald Nkangi, Rebecca Amongin, Ludoviko Zirimenya, Emily L. Webb, Alison M. Elliott, Gyaviira Nkurunungi

**Affiliations:** 1 Immunomodulation and Vaccines Focus Area, Vaccine Research Theme, MRC/UVRI and LSHTM Uganda Research Unit, Entebbe, Uganda; 2 University of Glasgow, Glasgow, UK; 3 Department of Infection Biology, London School of Hygiene and Tropical Medicine, London, UK; 4 International Statistics and Epidemiology Group, Department of Infectious Disease Epidemiology, London School of Hygiene and Tropical Medicine, London, UK; 5 Leiden University Center for Infectious Diseases (LU-CID), Leiden University Medical Center, Leiden, The Netherlands; 6 Department of Clinical Research, London School of Hygiene and Tropical Medicine, London, UK

**Keywords:** Immunology, Infection, Infectious diseases, Vaccines, Dysbiosis, Parasitic infection, Epidemiology

## Abstract

Vaccine responses are sometimes impaired in rural, low-income settings. Helminth-associated gut barrier dysfunction and microbial translocation (MT) may be implicated. We used samples from a trial of praziquantel treatment-effects on vaccine responses in *Schistosoma mansoni* (*Sm*)-endemic Ugandan islands, measuring intestinal fatty acid-binding protein 2 (I-FABP2), lipopolysaccharide-binding protein, anti-endotoxin core antibodies (EndoCab), soluble CD14 (sCD14) in plasma, and faecal lipocalin-2, occult blood (FOB), and calprotectin (fCAL), and evaluating their associations with baseline helminth infection, praziquantel treatment, and responses to BCG, yellow fever, typhoid, HPV, and tetanus-diphtheria vaccines. *Sm* associated positively with fCAL and FOB, hookworm with I-FABP2, and any helminth with EndoCab IgM, fCAL and FOB. *Sm* associated inversely with sCD14. Praziquantel treatment reduced all marker concentrations, significantly fCAL and FOB, implying that *Sm*-associated gut inflammation and MT is reversible. Associations of assessed markers with vaccine-specific responses were predominantly inverse. Interventions to improve gut barrier function may enhance vaccine responsiveness.

## Introduction

Vaccines have played a vital role in the control of infectious diseases; however, some exhibit impaired effectiveness and immunogenicity in tropical low-income countries (LIC) compared to temperate high-income countries (HIC)^1–7^. International^2,3^ and regional^1^ disparities are documented for BCG, with reduced efficacy and immunogenicity at lower latitudes. Other licensed vaccines, such as those against rotavirus^8^, cholera^9^ and yellow fever^4^ are similarly affected, as are investigational vaccines: the heterologous prime-boost Ebola virus vaccine regimen showed significantly lower vaccine-induced IgG responses in Senegalese compared to UK participants^7^. Furthermore, several studies in LICs show reduced vaccine immunogenicity among rural-dwelling individuals compared to their urban counterparts^10–12^. For example, in Ugandan school children receiving the same vaccination schedule in different geographical settings, we recently reported lower yellow fever, oral typhoid and tetanus vaccine-specific responses in rural, schistosomiasis-endemic and malaria-endemic settings, compared to a more urban setting^13^.

Geographical differences in vaccine effectiveness and immunogenicity remain incompletely understood. While genetic differences between populations^14^ or differential prior exposure to target or related pathogens^15,16^ may contribute, neither can explain the full extent of this disparity, as illustrated by vaccine response variations between proximate but environmentally diverse rural and urban LIC settings^13^, and international differences in vaccine responses for rare pathogens^7^. Environmental exposures appear to be the main drivers^17^. Evidence suggests that immunomodulation by chronic infections, such as parasitic helminths prevalent in tropical latitudes, has non-specific bystander effects in the mammalian host, including on vaccine responses^18^.

Helminths have been shown to alter host immunity; for example, by inducing a regulatory or Th2-type cellular phenotype^19^, an alternatively activated macrophage phenotype^20^ and immune exhaustion^21^, which separately, or concertedly, downregulate the Th1-type and inflammatory immune state required to augment vaccine-induced antibody or cytokine responses. Helminth modulation of vaccine responses might also be mediated by other host factors, such as altered gut microbiota, and, or, through increased translocation of microbial products into the systemic circulation^22–24^ following helminth-associated gut mucosal damage. Immunomodulation by translocated microbial products has been demonstrated in mouse models: infection with *Heligmosomoides polygyrus* led to heightened responses to exogenous Respiratory Syncytial Virus in the lung, through interaction with the gut microbiome, gut microbial translocation and increased type I interferon expression^25^.

We used the opportunity provided by our randomised trial in a helminth-endemic rural Ugandan setting with particularly high *Schistosoma mansoni* (*S. mansoni*) prevalence to determine associations between helminth infections and markers of gastrointestinal inflammation and microbial translocation, to investigate whether there are reversible effects of chronic *S. mansoni* infection on these markers, and to explore possible associations with vaccine-specific immune responses.

## Results

### High prevalence of helminth infection in Ugandan island schoolchildren

**Fig. 1:**
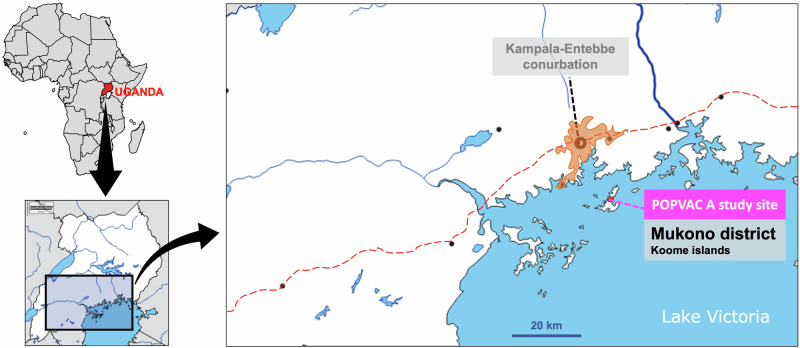
Study setting.

**Fig. 2:**
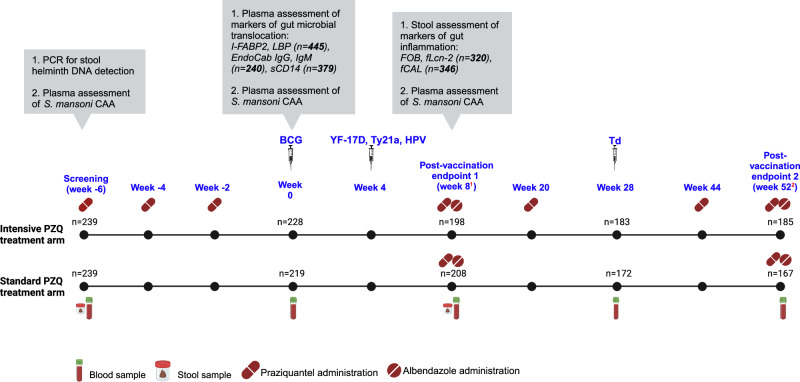
Study schedule. Samples were collected before vaccinations and/or anthelminthic treatment at relevant timepoints. Only stool and blood samples used for assessment of helminth infections and markers of gut inflammation and microbial translocation are shown. Praziquantel and albendazole were administered to all participants, following the protocol, independent of helminth infection status. Helminth infection status was determined retrospectively, after samples at relevant timepoints had been collected. BCG Bacille Calmette-Guérin, YF-17D Yellow Fever, Ty21a oral typhoid, HPV Human Papillomavirus, Td Tetanus-diphtheria, PZQ praziquantel, CAA circulating anodic antigen, I-FABP2 intestinal fatty acid-binding protein, LBP lipopolysaccharide (LPS)-binding protein, sCD14 soluble CD14, EndoCab anti-endotoxin core antibody, fCAL faecal calprotectin, FOB faecal occult blood, fLcn-2 faecal lipocalin 2. ^**1**^Primary endpoint following BCG, YF-17D, Ty21a, and HPV vaccination. ^**2**^Primary endpoint following Td vaccination; secondary endpoint following BCG, YF-17D, Ty21a, and HPV vaccination. Created in BioRender. Nkurunungi, G. (2025) https://BioRender.com/y64k537.

Between July 9 and August 14 2019, we recruited 478 schoolchildren into the POPulation differences in VACcine responses trial A (POPVAC A)^26^ in the rural helminth-endemic Koome islands of Lake Victoria, Uganda (Fig. 1). The current investigation used samples from POPVAC A to measure plasma markers of gut microbial translocation (intestinal fatty acid-binding protein 2 [I-FABP2], lipopolysaccharide-binding protein [LBP], anti-endotoxin core antibodies [EndoCab], soluble CD14 [sCD14]), and faecal markers of gut inflammation (lipocalin-2 [fLcn-2], occult blood [FOB], and calprotectin [fCAL]), evaluating their associations with baseline helminth infection, praziquantel treatment, and vaccine responses. Figure 2 shows the study schedule, further detailed in the methods section. Characteristics of study participants from whom we obtained data on at least one marker of gut inflammation or microbial translocation are shown in Table 1. The baseline prevalence of *S. mansoni* infection (circulating anodic antigen [CAA] ≥30 pg/ml) in this group was 40%, while 24% and 8% of participants were infected with *N. americanus* (hereinafter hookworm) and *S. stercoralis*, respectively. We detected infection with at least one of *S. mansoni*, hookworm or *S. stercoralis* in 58% of participants.

**Table 1:** Baseline characteristics of study participants

Demographics	
Age in years, median (IQR)	10 (11, 13)
Male sex, *n*/*N* (%)	261/449 (58.1)
**Helminth infections**, *n*/*N* (%)^**a**^	
*S. mansoni* (CAA ≥ 30 pg/ml)	177/446 (39.7)
*N. americanus* (PCR positive)	106/446 (23.8)
*S. stercoralis* (PCR positive)	36/446 (8.1)
Any helminth infection^b^	256/446 (57.6)
**Trial arm**^c^	
Intensively treated with praziquantel	228/449 (50.8)
Standard (not treated)	221/449 (49.2)

Table shows baseline characteristics for individuals with data on at least one marker of gut inflammation/microbial translocation.

*S. mansoni* Schistosoma mansoni, *N. americanus* Necator americanus, *S. stercoralis* Strongyloides stercoralis, *IQR* interquartile range, *CAA* circulating anodic antigen, *PCR* polymerase chain reaction

^a^*S. mansoni* CAA data presented are from week 0. For soil transmitted helminths, all PCR data are from the screening (week -6) timepoint; no significant change in prevalence was expected by week 0, as there was no albendazole treatment.

^b^Infection with any of S. mansoni (CAA), N. americanus (PCR) or S. stercoralis (PCR).

^c^Participants in the intensive arm were treated with praziquantel at two-week intervals prior to week 0, and then at week 8 and subsequent quarterly administrations until week 52. Standard arm participants received praziquantel treatment only following primary outcome assessment at week 8, with a second and final dose at week 52.

### Helminth infections are associated with gut inflammation and microbial translocation

**Fig. 3:**
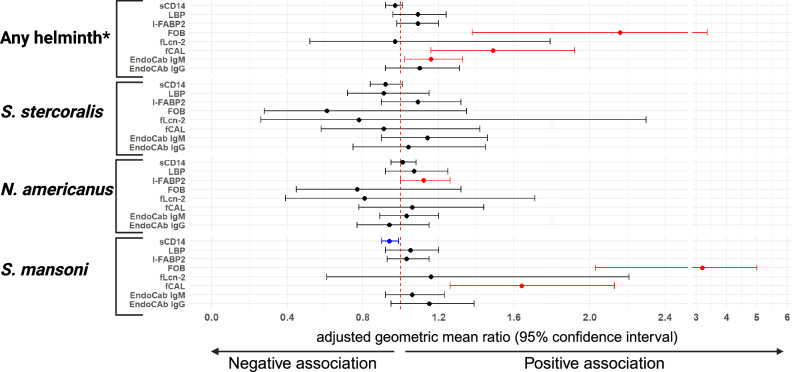
Effect of baseline helminth infections on levels of markers of gut microbial translocation and gut inflammation. Forest plot shows adjusted geometric mean ratios (aGMRs) and 95% confidence intervals (CIs) for associations between helminth infections and levels of markers of gut microbial translocation and gut inflammation. Analyses were adjusted for age and sex, and for *S. mansoni*, trial intervention arm (intensive vs standard praziquantel treatment). Reference category is the uninfected group. The asterisk refers to Infection with any of *S. mansoni*, hookworm or *S. stercoralis*. I-FABP2 intestinal fatty acid-binding protein, LBP lipopolysaccharide (LPS) binding protein, sCD14 soluble CD14, EndoCab anti-endotoxin core antibody, fCAL faecal calprotectin, FOB faecal occult blood, fLcn-2 faecal lipocalin 2.

*S. mansoni* infection was positively associated with fCAL, with an adjusted geometric mean ratio (aGMR) of 1.64, a 95% confidence interval (CI) ranging from 1.26 to 2.13, *p* < 0.001, and an empirical *p*-value after Monte Carlo permutations (to correct for multiple testing) of 0.001. *S. mansoni* was also positively associated with FOB (aGMR 3.19 [95%CI 2.03, 5.01], *p* < 0.001, *p*_corrected_ < 0.001), but inversely associated with sCD14 concentration (0.94 [0.90, 0.99], *p* = 0.018, *p*_corrected_ = 0.138). Hookworm infection was positively associated with I-FABP2 (1.12 [1.00, 1.26], *p* = 0.059, *p*_corrected_ = 0.397). Baseline infection with any of *S. mansoni*, hookworm or *S. stercoralis* was positively associated with EndoCab IgM (1.16 [1.02, 1.33], *p* = 0.024, *p*_corrected_ = 0.170), fCAL (1.49 [1.16, 1.92], *p* = 0.002, *p*_corrected_ = 0.024) and FOB concentrations (2.16 [1.38, 3.36], *p* = 0.001, *p*_corrected_ = 0.006) [Fig. 3 and Supplementary Table 1].

We further analysed associations between helminth infections and the assessed markers focussing on mono-infected individuals versus those not infected with any of *S. mansoni*, hookworm or *S. stercoralis* (Supplementary Fig. 1 and Supplementary Table 2). *S. mansoni* infection was positively associated with EndoCab IgM (1.20 [1.01, 1.42], *p* = 0.036, *p*_corrected_ = 0.264), fCAL (1.53 [1.12, 2.09], *p* < 0.001, *p*_corrected_ = 0.062) and FOB (3.16 [1.80, 5.55], *p* < 0.001, *p*_corrected_ < 0.001).

The *S. mansoni* CAA test lacks established thresholds to classify infection intensity as low, moderate, or heavy. To assess associations between *S. mansoni* infection intensity and concentrations of markers of gut microbial translocation and inflammation, we categorized individuals with CAA ≥ 30 pg/ml into the lowest, middle, and highest tertile groups, representing low, moderate, and heavy infection intensity, respectively (Supplementary Table 3a). Our analysis showed that geometric means of some markers (EndoCab IgG, fCAL, FOB) increased with increasing infection intensity (Supplementary Table 3b).

### Intensive praziquantel intervention reduces concentrations of markers of gut inflammation and microbial translocation

**Fig. 4:**
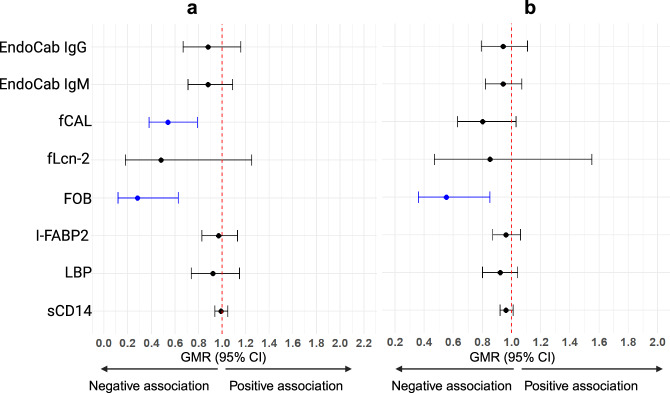
Impact of intensive praziquantel treatment on levels of markers of gut microbial translocation and gut inflammation. Forest plots show geometric mean ratios (GMRs) and 95% confidence intervals (CIs) for associations between praziquantel treatment and levels of markers of gut microbial translocation and gut inflammation, among (**a**) participants who were *S. mansoni* infected (CAA ≥ 30 pg/ml) at enrolment (week -6) in both standard and intensive intervention arms, but were *S. mansoni* negative (CAA < 30 pg/ml) in the intensive arm six weeks later (week 0), and (**b**) all randomised participants regardless of *S. mansoni* infection status at enrolment. The reference category for the linear regression model was the standard intervention arm. *Sm* *Schistosoma mansoni*, CAA circulating anodic antigen, I-FABP2 intestinal fatty acid-binding protein, LBP lipopolysaccharide (LPS)-binding protein, sCD14 soluble CD14, EndoCab anti-endotoxin core antibodies, fCAL faecal calprotectin, FOB faecal occult blood, fLcn-2 faecal lipocalin-2.

Figure 4, Supplementary Fig. 2 and Supplementary Table 4 show the impact of intensive versus standard praziquantel administration on concentrations of markers of gut inflammation and microbial translocation. In participants who were *S. mansoni* infected (CAA ≥ 30 pg/ml) at enrolment (week -6) in both standard (*n* = 124) and intensive (*n* = 131) intervention arms, but were *S. mansoni* negative (CAA < 30 pg/ml) in the intensive arm six weeks later (week 0, *n* = 64), intensive, compared to standard, praziquantel administration reduced the concentrations of gut inflammation and translocation markers, although only associations with fCAL (aGMR 0.57 [95% CI 0.39, 0.81], *p* = 0.002, *p*_corrected_ = 0.018) and FOB (0.27 [0.12, 0.63], *p* = 0.003, *p*_corrected_ = 0.019) reached statistical significance (Fig. 4a and Supplementary Table 4a). Comparable results were also observed for all randomised participants regardless of *S. mansoni* infection status at enrolment and at week 0 (Fig. 4b and Supplementary Table 4b).

### Associations between vaccine responsiveness and markers of gut inflammation and microbial translocation are predominantly inverse

**Fig. 5:**
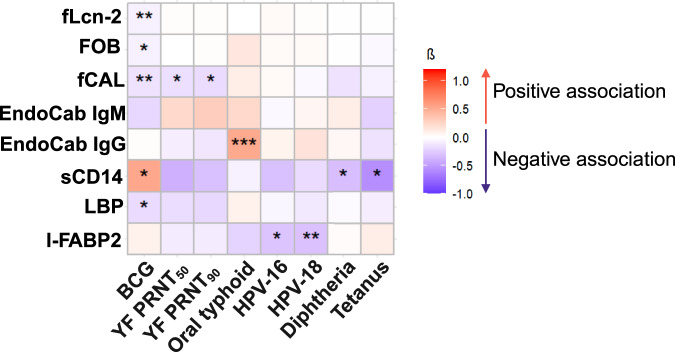
Associations between levels of markers of gut microbial translocation/gut inflammation and vaccine responses. Analyses are adjusted for age and sex. Asterisks represent significant associations. One asterisk represents associations at *p* < 0.05; two asterisks represent associations at *p* < 0.01; three asterisks represent associations at *p* < 0.001. β regression coefficient, I-FABP2 intestinal fatty acid-binding protein, LBP lipopolysaccharide (LPS)-binding protein, sCD14 soluble CD14, EndoCab anti-endotoxin core antibodies, fCAL faecal calprotectin, FOB faecal occult blood, fLcn-2 faecal lipocalin-2.

Building on our prior review^18^ and recent observational analysis in this study population^27^, which support a role for helminths in altering responses to some vaccines, as well as the above findings indicating that helminths may drive gut inflammation and microbial translocation, we next assessed relationships between concentrations of markers of gut inflammation and microbial translocation, and vaccine-specific responses measured at the POPVAC A trial primary endpoint (week 8, or, for Td, at week 52) [Fig. 5 and Supplementary Table 5]. There were inverse associations between the following markers and vaccine-specific responses: fLcn-2 (adjusted regression coefficient β −0.06 [95% confidence interval −0.09, −0.02], *p* = 0.001, *p*_corrected_ = 0.006), FOB (−0.06 [−0.10, −0.01], *p* = 0.015, *p*_corrected_ = 0.112), fCAL (−0.12 [−0.20, −0.04], *p* = 0.003, *p*_corrected_ = 0.025) and LBP (−0.15 [−0.28, −0.03], *p* = 0.017, *p*_corrected_ = 0.118) with BCG-specific IFN-ɣ; fCAL with yellow fever PRNT_50_ (−0.13 [−0.26, −0.005], *p* = 0.042, *p*_corrected_ = 0.293) and PRNT_90_ titres (−0.15 [−0.27, −0.03], *p* = 0.016, *p*_corrected_ = 0.127); sCD14 with diphtheria toxoid- (−0.27 [−0.51, −0.03], *p* = 0.030, *p*_corrected_ = 0.226) and tetanus toxoid-specific IgG (−0.47 [−0.95, 0.002], *p* = 0.051, *p*_corrected_ = 0.329); and I-FABP2 with HPV-16- (−0.24 [−0.44, −0.03], *p* = 0.025, *p*_corrected_ = 0.171) and HPV-18-specific IgG (−0.25 [−0.42, −0.07], *p* = 0.005, *p*_corrected_ = 0.041). Conversely, positive associations were observed between sCD14 and BCG-specific IFN-ɣ (0.44 [0.05, 0.83], *p* = 0.026, *p*_corrected_ = 0.178), and between EndoCab IgG and *S*. Typhi O:LPS-specific IgG (0.43 [0.21, 0.65], *p* < 0.001).

Considering vaccine-specific responses measured at the POPVAC A trial secondary endpoint (week 52, for BCG, yellow fever, oral typhoid and HPV vaccines), the only observed significant inverse associations were between sCD14 and HPV-16- (−0.79 [−1.50, −0.07], *p* = 0.031, *p*_corrected_ = 0.263) and HPV-18-specific IgG (−0.57 [−1.11, −0.04], *p* = 0.035, *p*_corrected_ = 0.258), while significant positive associations were observed between *S*. Typhi O:LPS-specific IgG and EndoCab IgG (0.51 [0.26, 0.76], *p* < 0.001) and fCAL (0.12 [0.01, 0.24], *p* = 0.041, *p*_corrected_ = 0.245) [Supplementary Table 6].

Supplementary Fig. 2 and Supplementary Table 7 show associations between concentrations of markers of gut inflammation and microbial translocation, and vaccine-specific responses, the latter represented as the absolute increase in vaccine-specific responses from baseline to the primary endpoint. Inverse associations were observed between sCD14 and diphtheria toxoid- (−0.31 [−0.54, −0.08], *p* = 0.009, *p*_corrected_ = 0.069) and tetanus toxoid-specific IgG (−0.49 [−0.96, −0.01], *p* = 0.046, *p*_corrected_ = 0.311), and between I-FABP2 and HPV-16- (−0.26 [−0.49, −0.03], *p* = 0.024, *p*_corrected_ = 0.166) and HPV-18-specific IgG (−0.28 [−0.49, −0.06], *p* = 0.011, *p*_corrected_ = 0.083). The only observed significant positive association was between sCD14 and *S*. Typhi O:LPS-specific IgG (1.03 [0.10, 1.96], *p* = 0.030, *p*_corrected_ = 0.213).

Lastly, we assessed associations between concentrations of markers of gut inflammation and microbial translocation, and protective tetanus toxoid-specific IgG levels (≥0.1 IU/ml) post Td vaccination, and seropositivity (≥4-fold increase in *S*. Typhi O:LPS-specific IgG) following Ty21a vaccination (Supplementary Table 8). In both cases, ≥90% of participants were protected or seropositive, and our analyses found no significant associations.

## Discussion

Our analysis examined relationships of markers of gut inflammation and microbial translocation with baseline helminth infections, praziquantel treatment, and vaccine-specific responses among schoolchildren from rural helminth-endemic Koome Islands of Lake Victoria. Our findings indicate that helminth infections, especially *S. mansoni*, drive intestinal inflammation and likely enhance microbial translocation. Praziquantel treatment appeared to reverse these *Schistosoma*-driven effects. Associations between the studied markers and vaccine responses were varied; however, higher levels of inflammation and microbial translocation were predominantly associated with reduced vaccine-specific responses.

In line with findings by Bustinduy et al. ^28^, infection with *S. mansoni* in our participants was associated with elevated levels of FOB and fCAL. Concentrations of these markers increased with increasing plasma *S. mansoni* CAA levels. Infection with any of the assessed helminths was positively associated with FOB, fCAL and EndoCab IgM, and infection with hookworm was positively associated with plasma I-FABP2 levels. These results support a role for *S. mansoni* and other intestinal helminths in driving gut inflammation and gut microbial translocation. For *S. mansoni*, extravasation of the parasite’s eggs through the intestinal wall into the lumen weakens the intestinal barrier, and formation of granulomas around eggs leads to disruption of intestinal architecture^29^, likely contributing to barrier dysfunction. This may alter composition of gut microbiota^30^: certain bacterial species may be more adept at colonizing the damaged gut and translocating across the compromised barrier.

Our findings suggest that the effects of intestinal helminths on gut inflammation and microbial translocation are reversible. Consistent with our findings, Bustinduy et al.^28^ reported a decrease in levels of the gut inflammation faecal markers fCAL and FOB following praziquantel treatment. Our study further observed a reduction in levels of systemic markers of gut microbial translocation after praziquantel administration. However, these reductions were not statistically different when comparing intensively treated with untreated (standard arm) participants. The more rapid post-treatment decline in faecal compared to systemic markers suggests that systemic markers may reflect a more complex physiological process influenced by factors beyond immediate gut barrier integrity. For example, plasma EndoCab, LBP and sCD14 represent downstream responses to LPS that has translocated across a damaged and/or leaky gut^31^.

Previous research attempting to establish a link between enteric dysfunction and the underperformance of vaccine responses has predominantly focussed on oral vaccines. Results have been inconsistent^32^, with studies showing both negative and positive associations. In a Bangladeshi cohort, sCD14 levels measured in 6-week-old infants were negatively correlated with both the immune response to the first dose of the oral polio vaccine and protection against rotavirus. However by 16 weeks, this correlation had changed to a positive one, but only for the polio vaccine^33^. In our study we found a general trend of inverse associations between various gut microbial translocation and inflammation markers and vaccine responses measured four weeks (for HPV, Ty21a, YF-17D), eight weeks (for BCG) or 24 weeks (for Td) post-vaccination, indicating that gut inflammation and microbial translocation could contribute to attenuation of vaccine-specific immune responses. As also discussed by Church et al.^32^, while it is possible that factors other than gut inflammation and microbial translocation explain the reduced effectiveness of vaccines in low-income countries, the notable overlap between intestinal damage by helminths and poor vaccine performance is striking, as is the significantly greater extent of intestinal damage in children from these regions compared to those in high-income countries.

We observed a significant positive association between oral typhoid vaccine response and EndoCab IgG; this is probably explained by potential cross-reactivity in the assay with the actual typhoid-specific antigens, as the vaccine consists of live (attenuated) bacteria. The reason behind the positive associations between sCD14 and vaccine responses for BCG and oral typhoid remains unclear.

Helminth-associated bystander effects on immune responses at distal sites are well documented. This includes immune responses to vaccines. Our recent review found that helminths are generally associated with reduced vaccine responses, although the magnitude of this effect varied across different vaccine types and was characterized by significant heterogeneity^18^. Our recent observational analysis also supports a role for helminths in altering responses to some vaccines^27^. One hypothesised mechanism involves helminth-induced gut mucosa damage and subsequent translocation of microbial products into the systemic circulation. Increased translocation leads to increased numbers of antigen-presenting cells (such as dendritic cells) carrying gut microbial antigens. Therefore, it is plausible that the immune system is diverted from mounting an optimal vaccine response while focussed on combating systemic microbial invasion^34^. Furthermore, systemic increase in epitopes (encoded by gut microbiota) that are cross-reactive with epitopes from target vaccine antigens might alter the response to the vaccine. Conversely, translocated products such as LPS could provide adjuvant effects to vaccines, and metabolites such as short chain fatty acids derived from gut microbiota could enhance antibody and cellular responses—although we found modest evidence of positive associations in our investigation.

The strengths of our study include its randomized trial design to assess the effect of praziquantel intervention on gut inflammation and translocation, and the inclusion of a diverse portfolio of vaccines. This design allowed us to explore the potential effects of gut microbial translocation and inflammation on various vaccine types, enhancing the robustness and breadth of our findings. However, while plasma markers were assessed at the week 0 pre-vaccination timepoint, faecal markers were assessed at week 8, because there was no stool sample was collected at week 0. It is possible that vaccinations administered and other factors such as new helminth infections between week 0 and week 8 might have affected relationships of the assessed markers with baseline helminth infections and vaccine responses.

In conclusion, we show that *Schistosoma mansoni* drives gut inflammation and potentiates microbial translocation, that this effect is potentially reversible with praziquantel treatment, and that gut barrier dysfunction might alter vaccine responses. Our findings call for further studies to explore the effect of elevated gut microbial translocation on the immune system, particularly on phenotypes that have been hypothesised to alter vaccine responsiveness, such as immune cell exhaustion and senescence^35^. The interplay between the gut microbiome and related metabolome, intestinal helminths, microbial translocation and consequently vaccine responses, should also be studied. Further interventional efforts such as administration of probiotics to reduce gut translocation^36^, and pre-emptive anthelminthic treatment to restore gut barrier function, might contribute to optimal vaccine responsiveness in tropical low-income settings.

## Materials and methods

### Ethics statement

Informed written assent was obtained from all participants and written informed consent from their respective parents or guardians. Ethical approval was granted by the Uganda Virus Research Institute Research Ethics Committee (references GC/127/18/09/680 GC/127/19/05/664), the London School of Hygiene and Tropical Medicine Observational/Interventions Research Ethics Committee (reference 16032), the Uganda National Council for Science and Technology (reference HS2486), and the Uganda National Drug Authority (reference CTA0093).

### Study design and participants

We used samples and data from 9-17-year-old individuals participating in the POPulation differences in VACcine responses trial A (POPVAC A, registration ISRCTN60517191)^26,37^ in the rural helminth-endemic Koome islands of Lake Victoria, Uganda (Fig. 1). These islands are located approximately 35 km from the closest mainland, comprise mainly fishing communities, and have particularly high *Schistosoma mansoni* (*S. mansoni*) prevalence^26,38^. POPVAC A was an open-label individually randomised, controlled trial of intensive versus standard praziquantel treatment against *S. mansoni* (Fig. 2), designed to investigate the hypothesis that *S. mansoni* infection downmodulates responses to unrelated vaccines, and that this can be reversed by intensive anthelminthic treatment.

Participants in the intensive arm received three doses of praziquantel (approximately 40 mg/kg, determined by the WHO height pole) at two-week intervals prior to the initial trial vaccination (week 0), followed by another dose at week 8 and subsequent quarterly administrations until week 52. Conversely, the standard arm participants commenced praziquantel treatment following primary outcome assessment at week 8, with a second and final dose at the study conclusion (week 52). All study participants also received single-dose albendazole (400 mg) at weeks 8 and 52, in line with Uganda’s Ministry of Health guidelines for helminth control.

The vaccination schedule is summarised in Fig. 2. Trial participants received the following vaccines: live parenteral (BCG, from Serum Institute of India, and yellow fever—YF-17D, from Sanofi Pasteur, France), live oral (typhoid—Ty21a; Vivotif, from PaxVax, UK), virus-like particle (Human Papillomavirus, HPV, from Gardasil, Merck & Co, USA) and toxoid (Tetanus/Diphtheria, Td, from Serum Institute of India) vaccines. The vaccination regimen comprised three primary immunization days at weeks 0, 4, and 28. HIV positive individuals were excluded from the trial.

### Laboratory assessment of vaccine-specific responses

Primary outcomes for the parent POPVAC A trial, and for the current investigation, were vaccine-specific responses at week 8, or, for Td, at week 52. Specifically, we assessed BCG-specific interferon (IFN)-gamma responses 8 weeks post-BCG vaccination, YF-17D neutralizing antibody titres at 4 weeks post-YF-17D vaccination, *Salmonella* Typhi O-lipopolysaccharide (O:LPS)-specific IgG levels at 4 weeks post-Ty21a vaccination, HPV type 16 and type 18 L1 protein-specific IgG concentrations at 4 weeks post-HPV, and tetanus and diphtheria toxoid-specific IgG levels at 24 weeks post-Td vaccination. Secondary vaccine response outcomes for the POPVAC A trial were vaccine-specific responses at week 52, for BCG, YF-17D, Ty21a and HPV. These responses were assessed as previously described^26^. Details are further documented below.

#### Ex vivo interferon-γ ELISpot assays to quantify BCG-specific responses

To quantify BCG-specific responses, we conducted ex vivo interferon (IFN)-γ ELISpot assays, using a Human IFN-γ (ALP) ELISpot Flex kit (Mabtech, Sweden) and multiscreen-IP 0.45μm filter 96-well plates (Merck Millipore). ELISpot plates were coated overnight at 4 °C with 50 μl of anti-IFN-γ capture antibody (15 μg/ml) dissolved in 0.05 M carbonate-bicarbonate buffer (Sigma Aldrich). The plates were then washed 5 times with sterile 1X PBS (Sigma Aldrich), and blocked (2–5 hours, 37 °C) by adding 100 μl/well of R10 medium (10% fetal bovine serum [Sigma Adrich] in RPMI 1640 medium [Thermofisher scientific] supplemented with L-glutamine, streptomycin, HEPES buffer and penicillin [all from Life technologies, UK]). During plate blocking, peripheral blood mononuclear cells (PBMCs) were isolated from heparinised whole blood by density gradient centrifugation with Histopaque® (Sigma Aldrich). For each study sample, PBMCs (300,000 per test well) were stimulated in duplicate for 18–20 hours at 37 °C, 5% CO_2_, with BCG (Moscow strain, Serum Institute of India) at a concentration of 200,000 colony forming units per ml, or left unstimulated. Staphylococcal enterotoxin B (SEB; Sigma Aldrich) was used at a final concentration of 10 μg/ml as a positive control, and a 1:1 mix of the 6-kDa early secretory antigenic target and 10-kDa culture filtrate protein (ESAT-6 and CFP-10 recombinant proteins from BEI Resources, USA) used at a final concentration of 2.5 μg/ml for exploratory assessment of tuberculosis infection. Following the 18–20 hour incubation, plates were washed 5 times with 200ul/well PBS containing 0.05% Tween 20 (Sigma Aldrich) and incubated for 2 hours at room temperature with 50 μl per well of a 1/1000 PBS dilution of biotin anti-IFN-γ antibody from the ELISpot kit. After another washing step with PBS-0.05% Tween 20, plates were incubated for 1 hour at room temperature with 50 μl per well of a 1/1000 PBS dilution of a streptavidin-alkaline phosphatase conjugate from the ELISpot kit. Plates were washed, developed for 3–10 minutes with 50 μl per well of 5-Bromo-4-chloro-3-indoxyl phosphate/Nitro blue tetrazolium (BCIP/NBT; Europa Bioproducts), and the reaction stopped by washing the plate under tap water. Plates were allowed to dry in the dark at room temperature and read using an ELISpot reader (Autoimmun Diagnostika Gmbh iSpot, Strassberg, Germany) running AID ELISpot software v.7.0. Spot-forming units (SFUs) per well were manually verified to remove artefacts.

We performed QC through a number of steps: 1) for each sample, we checked to ascertain whether the PBMC isolation procedure was conducted within eight hours after sample collection; 2) we inspected each ELISpot plate visually for quality and completeness of labelling (sample IDs, date, time point and antigens) and compared the plate picture to the exported spot count spreadsheet to ensure the correct data had been exported; 3) we checked the calculated data in the exported database to ensure the background subtraction, average of duplicate wells and multiplication up to spot forming units per million PBMC had been performed correctly; 4) we assessed whether the unstimulated well and SEB well controls for each assay were within the accepted range.

Results were reported as SFUs per a million PBMCs, calculated sequentially by 1) subtracting mean SFUs of unstimulated wells from mean SFUs of duplicate antigen wells, and 2) correcting for the number of PBMCs per well (300,000). Samples that had more than 83.3 SFUs per a million PBMCs in the unstimulated well were considered invalid and not included in the final analysis.

#### Yellow Fever plaque reduction neutralizing test (PRNT)

A plaque reduction assay as described by Beaty et al.^39^ was used. Briefly, Vero cells at a concentration of 65,000 cells/ml were seeded into 6-well plates (Greiner Bio-One GmbH, Germany) at a volume of 3 ml/well. Cells were cultured in growth medium (1X Eagle’s Minimum Essential Medium, 8% heat inactivated fetal bovine serum, 100 units penicillin/streptomycin, gentamycin 50 mg/ml and fungizone 1 mg/ml) at 37 °C (with 5% CO_2_) for 3–4 days. Culture medium was then removed from the cell monolayer by dumping. Test plasma were inactivated at 56 °C for 30 min to remove complement factor, serially (two-fold) diluted from 1:10 to 1:20480 in BA-1 diluent (10X M199 Hanks’ Salts without L-Glutamine, 5% Bovine Serum Albumin, 1 M TRIS-HCL pH 7.5, L-Glutamine, sodium bicarbonate 7.5%, 100X penicillin/streptomycin, 1000X fungizone in sterile water), and mixed with approximately 200 Plaque Forming Units (PFU) of a reference YF-17D virus preparation. The plasma-virus mixture (0.1 ml) was added to the confluent monolayer of Vero cells in each well and incubated at 37 °C (with 5% CO_2_) for 1 hour. The first overlay medium (comprising Miller’s 2X Yeast Extract-Lactalbumin hydrolysate medium, 10X Earle’s Buffered Salts Solutions, 2% fetal bovine serum, 1000X fungizone, 1000X Gentamycin, and 2% low-melting agarose) was added, 3 ml per well, and allowed to solidify for 30 minutes at room temperature. The plates were incubated at 37 °C with 5% CO_2_ for 4 days. To stain cell layers, a Neutral Red dye (Sigma Aldrich) second overlay was added, 2 ml per well, and allowed to solidify for 30 minutes at room temperature. After this second overlay, plates were incubated at 37 °C in 5% CO_2_ for 2 days: plaques were counted first on day 1 and the final score documented on day 2 to establish 50% and 90% neutralization titres. Back titration plates were established to ensure infectivity of cell monolayer and standardization of virus to 200 PFU/0.1 ml. Neutralisation titres <1:10 were considered negative. Titres of 1:10 were interpreted as borderline. The PRNT antibody titres presented refer to the reciprocal of the last plasma dilution that reduced by 50% (PRNT50) or 90% (PRNT90) the number of virus plaque clusters infected by 100 PFU/0.1 ml of the reference 17D virus preparation.

For quality control, we used a high titre positive control (PC), with the last six titre dilution range, from 640 to 20480. So long as the PC titre was within the expected range and did not vary by greater than 4-fold, the assay passed quality control. Furthermore, we ran back-titrations of the virus inoculum (standardised to 200 PFU/0.1 ml) to determine the end-point specimen antibody titre at 50% or 90% neutralisation. The number of virus plaques infected at 50% neutralisation and at 90% neutralisation were expected to be within an approximate range of 25–100 and 5–20, respectively.

#### Detection of plasma IgG against Salmonella Typhi O-lipopolysaccharide (O:LPS)

Specific IgG to *S. Typhi* O-lipopolysaccharide (O:LPS) was measured by an in-house ELISA. Nunc Maxisorp 96-well plates (Thermo Fisher) were coated overnight at 4 °C with 50 μl/well of 10 μg/ml of *S*. Typhi O:LPS (Sigma L2387) in bicarbonate (Na_2_CO_3_ + NaHCO_3_) buffer (0.1 M, pH 9.6). Plates were washed with phosphate-buffered saline (PBS 1X)-Tween 20 (0.05%) solution, blocked with 200 μl of 5% skimmed milk diluted in PBS-Tween 20 for 1 hour at room temperature (RT), washed again and incubated for 2 hours at RT with 50 μl of test plasma samples (diluted 1/320 with 1% skimmed milk in PBS-Tween 20) and two-fold serially diluted standard sera (top concentration 20 EU/ml). Standards were derived from a pooled sample generated from sera of known O:LPS-specific IgG titres, kindly provided by the Oxford Vaccine Centre Biobank. These sera had been collected from the highest responders to O-antigen following challenge with *S*. Typhi in a controlled human infection study^40^. Following test and standard sample incubation, plates were washed and O:LPS-specific IgG binding detected by incubating the plates for 1 hour at RT with goat anti-human IgG-horseradish peroxidase conjugate (Insight Biotechnology, UK), diluted 1/6000 in 1% skimmed milk–PBS-Tween 20. Plates were washed and developed by addition of 100 μl of o-phenylenediamine (Sigma-Aldrich) and reactions stopped after 5 minutes with 30 μl of 2 M Sulphuric acid. Optical density (OD) values were measured at 490 nm (reference wavelength 630 nm) on a 96-well plate ELISA reader (BioTek ELx808, USA). Nominal ELISA units (EU/ml), representing O:LPS-specific IgG titres, were interpolated from standard curves using a five-parameter curve fit using Gen5 data collection and analysis software (BioTek Instruments Inc, Vermont, Winooski, USA).

#### Detection of plasma IgG against Human Papillomavirus type 16 (HPV-16) and HPV-18

Anti-HPV-16 and HPV-18 IgG concentrations were measured by ELISA, as previously described^41–44^. Nunc Maxisorp 96-well plates (Thermo Fisher) were coated with 100 µl of HPV-16 L1 virus-like particles (VLP) at a concentration of 2.7 µg/ml, or with HPV-18 L1-L2 VLP at a concentration of 2 µg/ml and incubated at 4 °C overnight. Plates were washed three times with a 1X phosphate-buffered saline (PBS)-Tween 20 (0.25%) solution, and blocked for 90 minutes at room temperature (RT) with 4% skimmed milk diluted in a 1X PBS-0.25% Tween 20 solution. The plates were washed three times and and incubated (with gentle shaking) for 1 hour at RT with 100 μl of test plasma samples, assay controls and two-fold serially diluted standard sera. Pre-vaccination test plasma samples were diluted 1/50 (HPV-16 assay) or 1/200 (HPV-18 assay) with blocking buffer, while post-vaccination plasma samples were diluted 1/400 for both HPV-16 and HPV-18 assays. Standard sera were used at a top concentration of 1.28 EU/ml and 8.2425 EU/ml for HPV-16 and HPV-18 assays, respectively. Following test and standard sample incubation, plates were washed four times and further incubated for 1 hour at RT with peroxidase-labeled goat anti-human IgG (KPL, Gaithersburg, Maryland). Plates were then developed with a tetramethylbenzidine substrate solution (KPL, Inc.) for 25 minutes in the dark at room temperature. Next, the reaction was stopped by adding 100 μl of 0.36 N H_2_SO_4_ to each well. Optical density (OD) values were measured at 450 nm (reference wavelength 630 nm) on a 96-well plate ELISA reader (BioTek ELx808, USA). Nominal ELISA units (EU/ml), representing HPV-16 L1 VLP- and HPV-18 L1-L2 VLP-specific IgG titres, were interpolated from standard curves using a five-parameter curve fit using Gen5 data collection and analysis software (BioTek Instruments Inc, Vermont, Winooski, USA).

For quality control (QC), the acceptable R2 for the standard curve was ≥ 0.990 and the average optical density (OD) range of the top standard was 2.0-4.0. The acceptable OD of the last (8th) standard dilution was ≤0.25. The percentage difference in ODs between standard dilutions (i.e. n and n + 1 dilution) was expected to be ≥ 0.3. Plates were repeated if they failed to meet these standard curve criteria. The calculated negative control cut-off was 4 ± 3 EU/ml and 60 ± 10 EU/ml for HPV-16 and HPV-18, respectively. Plates whose negative control concentration was above the cut-offs were repeated. The calculated positive control concentration was 450 ± 10 EU/ml and 4500 ± 10 EU/ml for HPV-16 and HPV-18 respectively. Background signal was measured by a blank whose OD was expected to be ≤0.05. Higher ODs indicated assay contamination and plates were repeated.

#### ELISA measurement of anti-diphtheria and anti-tetanus IgG

Nunc Maxisorp 96-well plates (Thermo Fisher) were coated with 50 μl of either 2 Lf units per ml of diphtheria toxoid (NIBSC product code 13/212) per ml or 0.56 Lf units per ml of tetanus toxoid (NIBSC product code 02/232) in Na_2_CO_3_/NaHCO_3_ buffer (0.1 M, pH 9.6) overnight at 4 °C. Plates were washed with 0.05% Tween 20 in 1X phosphate-buffered saline (PBST) and blocked for 1 hour with 5% skimmed milk powder in PBST at 37 °C. The plates were washed four times and incubated for 2 hours at 37 °C with 50 μl of test plasma samples, and serially diluted WHO International Standard anti-toxins for diphtheria (NIBSC 10/262) or tetanus (NIBSC 13/240). Samples and standards were prepared in PBST + 1% skimmed milk (assay buffer). Pre-vaccination test samples were added at a dilution of 1/150, while post-vaccination plasma samples were added at a dilution of 1/300 in assay buffer. The standards were used at a top concentration of 3 IU/ml and 0.125 IU/ml for the tetanus and diphtheria assays, respectively. Plates were washed four times and incubated for 1 hour at 37 °C with 50 μl of polyclonal rabbit anti-human IgG HRP-conjugate (Agilent Dako, CA, USA) diluted 1/3000 in assay buffer. After another washing step, plates developed by adding 100 μl/well of o-phenylenediamine (Sigma-Aldrich) and reactions stopped after 5 minutes with 25 μl/well of 2 M sulphuric acid. Optical density (OD) values were measured at 490 nm (reference wavelength 630 nm) on a 96-well plate ELISA reader (BioTek ELx808, USA). Tetanus and diphtheria toxoid-specific IgG concentrations (IU/ml) were interpolated from standard curves using a five-parameter curve fit using Gen5 data collection and analysis software (BioTek Instruments Inc, Vermont, Winooski, USA).

### Laboratory assessment of helminth infections

Helminth infection status was assessed retrospectively, after samples at all study timepoints had been collected.

*S. mansoni* infection was determined by measurement of plasma circulating anodic antigen (CAA) using the up-converting phosphor lateral flow (UCP-LF) assay with a SCAA20 test format, with a 30 pg/ml positivity threshold^45^. This limit of detection was previously determined by spiking CAA (standard series) in negative serum from non-endemic healthy individuals and analyzed against a large set of confirmed CAA negative controls from different endemic regions. Based on this, quality control was conducted on test materials provided for the study to guarantee a lower limit of detection of 30 pg/ml. All samples above this level (≥30 pg/ml) are regarded as CAA-positive. For the current study, human negative serum was spiked with a known concentration of CAA standard (100,000 pg/ml) and diluted up to eight standard points, with two negative controls. These were used to generate a standard curve to quantify the plasma sample CAA levels. Previous work has shown that there is no difference between standard curves with spiked CAA in serum and in plasma^45^. Therefore, plasma and standards (50 µl) were extracted with an equal volume of 4% w/v trichloroacetic acid (TCA; Merck Life Science NV, the Netherlands), vortexed and incubated at ambient temperature for five minutes. Thereafter, samples and standards were briefly vortexed and spun at 13,000 g for five minutes. The resulting supernatant (20 µl) was added to the wells containing 100 ng dry UCP particles^46^ (400 nm Y2O2S:Yb3 + , Er3 + ) coated with mouse monoclonal anti-CAA antibodies^47^ hydrated with 100 µl of high salt lateral flow buffer (HSLF: 200 mM Tris pH8, 270 mM NaCl, 0.5% (v/v) Tween-20, 1% (w/v) BSA). These were incubated for one hour at 37 °C while shaking at 900 rpm. The CAA lateral flow strips^48^ were labeled with the standard and sample identifications and then placed in the wells on the UCP plate. The samples and standards were allowed to flow and left to dry overnight. The strips were then analysed using the Labrox Upcon reader (Labrox Oy, Turku, Finland). The test line signals (T; relative fluorescent units, peak area) were normalized to the flow control signals (FC) of the individual strips and the results were expressed as ratio values.

Multiplex polymerase chain reaction (PCR) using DNA extracted from stool was used to retrospectively assess infection with *Necator americanus* (hookworm) and *Strongyloides stercoralis*. Stool samples stored at −80 °C in 95% molecular grade ethanol were retrieved and thawed at room temperature. Total DNA (and hence helminth DNA, if present) was extracted from the stool samples using the Fast DNA Spin Kit for Feces (catalogue number 116570200, MP Biomedicals Germany GmbH) to determine (using multiplex real-time PCR) *Strongyloides stercoralis* and hookworm (*Necator americanus*) infections. The DNA extraction procedure was conducted with minor changes to the manufacturer’s instructions as follows: samples were left to thaw at room temperature (RT) and then vortexed for five seconds to homogenise the ethanol-stool mixture. The homogenized mixture (0.5 ml) was transferred into a safe-lock microcentrifuge (Eppendorf®) tube and centrifuged at 13000 rpm for 3 minutes to get rid of the ethanol. The pellet was re-suspended in a 2 ml lysing matrix E tube, containing 825 μl of sodium phosphate buffer and 275 μl of PLS buffer. For solid samples, 300–500 mg of stool were transferred to the lysis tube using a sterile loop. The samples were centrifuged for 5 minutes at 14000 g and the supernatant was decanted. 978 μl Sodium Phosphate Buffer and 122 μL MT Buffer were added to the lysing matrix E tube and vortexed briefly to mix the contents. The samples were then homogenised in the FastPrep® 24 instrument (MP Biomedicals Germany GmbH) at setting 6.0 m/s for 40 seconds and thereafter centrifuged at 14000 g for 5 minutes. The supernatant was then transferred to a clean 2.0 ml centrifuge tube and 250 μl of PPS solution was added. The samples were then shaken vigorously to mix, incubated at 4 °C for 10 minutes, and centrifuged at 14000 g for 2 minutes. During the centrifugation step, we added 1 ml of binding matrix solution to a clean 15 ml conical tube. The supernatant was then transferred to the 15 ml tube containing the binding matrix. These were mixed gently by hand for 3 to 5 minutes. The samples were then centrifuged at 14000 g for 2 minutes and the supernatant discarded. The binding mixture pellet was then washed by gently re-suspending it in 1 mL Wash Buffer #1. Two spins were performed by first transferring 600 μL of the binding mixture to a filter tube and centrifuged at 14000 g for 1 minute. The catch tube was then emptied, and the remaining binding mixture was added to the filter tube and centrifuged as before. The catch tube was emptied again and 500 μL of prepared Wash Buffer #2 (concentrated salt wash solution, reconstituted with absolute molecular grade ethanol) was added to the filter tube and re-suspended by gently pipetting up and down to dislodge the pellet. The samples were centrifuged at the same speed for 2 minutes and the flow-through discarded. The samples were centrifuged again for 2 minutes to extract residual ethanol from the binding matrix and dry the sample. The filter bucket was transferred to a clean 1.9 mL Catch Tube and 100 μL TES elution buffer added. The tube was stirred with a pipette tip to resuspend the pellet. The samples were then centrifuged for 2 minutes to elute purified DNA into the clean catch tube. The samples were stored at −20 °C until used for PCR (below). The multiplex real-time PCR was adapted from existing procedures^49,50^. Below are the specific forward (F) and reverse (R) primers and TaqMan® probes that were used. *Necator americanus:* Na58F:5’-CTGTTTGTCGAACGGTACTTGC-3’; Na158R: 5’- ATAACAGCGTGCACATGTTGC-3’; Nec-2-FAM (MGB): FAM-5’-CTGTACTACGCATTGTATAC-3’-XS; Nec-3-FAM (MGB): 5’-CTGTACTACGCATTGTATGT-3’. *Strongyloides stercoralis:* Stro18S-1530F: 5’-GAATTCCAAGTAAACGTAAGTCATTAGC-3’; Stro18S-1630R: 5’-TGCCTCTGGATATTGCTCAGTTC-3’; Stro-4-TRBhq2-VIC- 5’-ACACACCGSCCGTCGCTGC-3’. Phocine herpes virus (PhHV) DNA, extracted from the Phocine herpes virus (kindly provided by Dr. Martin Schutten, Erasmus Medical Center, Rotterdam, the Netherlands), was included in the PCR master mix, thus distributed to all reaction wells as an internal control to check for PCR inhibition. The PhHV forward primer PhHV-267s (5’-GGGCGAATCACAGATTGAATC-3’), reverse primer PhHV-337as (5’-GCGGTTCCAAACGTACCAA-3’) and probe PhHV-305tq (Cy5-5’ TTTTTATGTGTCCGCCACCATCTGGATC-3’-BHQ2) were used for Phocin herpes virus DNA detection. A positive pool was included on the plate for every run as a test control. The positive pool was made up of a mixture of DNA from samples (from among the study samples) that were highly positive for *N. americanus* on Kato-Katz, and for *S. stercoralis* by PCR (conducted on samples from a previous study). The amplification conditions were 15 minutes at 95 °C, 50 cycles of 15 seconds at 95 °C, 30 s at 60 °C and 30 s at 72C. DNA amplification, detection and data analysis were attained with the ABI 7500 Fast Real time machine and 7500 Fast systems software version 1.5.1.

### Laboratory assessment of markers of gut inflammation and microbial translocation

Plasma samples collected at week 0 (and stored at −80 °C) were retrospectively assessed for intestinal fatty acid-binding protein 2 (I-FABP2), lipopolysaccharide (LPS)-binding protein (LBP), anti-endotoxin core antibodies (EndoCab) and soluble CD14 (sCD14). Additionally, stool samples collected at week 8 (and stored at −80 °C) were retrospectively assessed for faecal lipocalin-2 (fLcn-2), faecal occult blood (FOB) and faecal calprotectin (fCAL). Faecal markers were not assessed at week 0 because there was no stool sample at that timepoint (Fig. 1).

I-FABP2, a protein involved in the intracellular transport of fatty acids and other lipophilic substances, is abundant in intestinal epithelial cells^51^, specifically in enterocytes, and leaks into the circulation following intestinal injury^52,53^. We used it as a plasma marker of gut wall integrity loss and inflammation. LBP recognises and responds to bacterial infections in the blood through binding bacterial LPS, presenting it to the cell surface receptor CD14. Therefore, we selected LBP, the soluble form of CD14 (the LPS co-receptor) and EndoCab IgM and IgG (antibodies targeting the core glycolipid component of LPS) as markers of gut microbial translocation^54,55^ and to reflect responses to LPS. These plasma markers were measured by enzyme-linked immunosorbent assay (ELISA) using commercial kits: I-FABP2, LBP, sCD14 (R&D Systems Inc., USA), EndoCab (HyCult Biotech, The Netherlands). We also selected fLcn-2, a protein secreted by neutrophils and involved in the immune response to bacterial infections through binding molecules (siderophores) that bacteria need to acquire iron^56–58^, FOB^28^, and fCAL, a calcium-binding protein found in neutrophils and released during inflammation in the gastrointestinal tract^28,59^. fLCn-2, FOB and fCAL are all recognised as markers of intestinal inflammation. To measure these faecal markers, we used a stool preparation kit from Immundiagnostik AG (catalogue number K 6998SAS, Bensheim, Germany) to homogenise 15 mg of stool with 1.5 ml of extraction buffer, obtaining a 1:100 dilution. Commercial ELISA kits were employed to assess fLcn-2 (R&D Systems, Inc., USA) and FOB (Immundiagnostik AG, Germany) concentrations in this stool suspension without any additional dilution step. However, to measure fCAL (Immundiagnostik AG, Germany), the stool suspension was further diluted 25-fold.

### Statistical analysis

Statistical analyses were conducted in Stata 18.0 (College Station, Texas, USA). Data visualisation was done in R (version 4.1.1; R foundation for Statistical Computing, Vienna, Austria) via the RStudio interface (RStudio, Inc. Boston, USA). Most raw concentrations of markers of translocation and inflammation, and vaccine response data, showed skewed distributions; therefore, log_10_(concentration +1)-transformed data were used for analyses. For analyses involving binary exposure variables (such as helminth infection status and trial intervention arm), regression coefficients were back-transformed to obtain geometric mean ratios (GMRs) and 95% confidence intervals (CIs).

Linear regression was used to evaluate associations between concentrations of the markers and helminth infection status and *S. mansoni* infection intensity, adjusting for age and sex. For associations with *S. mansoni* infection, we additionally adjusted for trial intervention arm (intensive vs standard praziquantel treatment).

Linear regression was also used to investigate the impact of intensive versus standard praziquantel treatment for schistosomiasis on levels of gut inflammation/translocation markers. Comparisons were conducted in two major analysis groups: **1)** participants who were *S. mansoni* infected (CAA ≥ 30 pg/ml) at enrolment (week -6) in both standard and intensive intervention arms, but were *S. mansoni* negative (CAA < 30 pg/ml) in the intensive arm six weeks later (week 0) – to investigate the effect of parasite removal on markers of gut inflammation/microbial translocation; **2)** all randomised participants regardless of *S. mansoni* infection status at enrolment. Analyses for group 2 did not adjust for covariates, as this was a randomised comparison. However, for comparisons in analysis group 1, we adjusted for age and sex.

Finally, we conducted linear regression analyses to evaluate associations between markers of gut inflammation or microbial translocation and responses to the portfolio of vaccines administered in the POPVAC trials, adjusting for age and sex. Vaccine responses were analysed in two ways: **1)** as the response at the POPVAC trial primary endpoint (week 8, or, for Td, at week 52), and **2)** as the absolute increase in response from baseline (pre-vaccination) to the primary endpoint. We further conducted logistic regression analysis to assess associations with protective antibody levels (for tetanus, IgG ≥0.1 IU/ml^60^) and seroconversion rates (for Ty21a, ≥4 fold increase over baseline^61^) following vaccination. All study participants had PRNT_50_ titres ≥10 four weeks post-YF-17D vaccination and were considered seropositive^62^. There are no recognised cut-offs for protection following BCG, HPV and diphtheria vaccination. For each vaccine-specific outcome, we only included in the analysis participants who received the corresponding vaccine; for Ty21a response, we only included participants who received at least one of the three Ty21a vaccine doses.

The above statistical tests were additionally subjected to Monte Carlo permutations^63^ based on 1000 permutations, to generate empirical *p*-values accounting for the fact that multiple markers were tested in each analysis. For completeness, we report *p* values both unadjusted and adjusted for multiple testing.

## Supplementary information

Supplementary Information

## Data Availability

The de-identified individual participant data that underlie the results reported in this article are stored in a non-publicly available repository (LSHTM Data Compass), together with a data dictionary. Data are available on request via 10.17037/DATA.00004505. Researchers who would like to access the data may submit a request through LSHTM Data Compass, detailing the data requested, the intended use for the data, and evidence of relevant experience and other information to support the request. The request will be reviewed by the corresponding authors in consultation with the MRC/UVRI and LSHTM data management committee, with oversight from the UVRI and LSHTM ethics committees. In line with the MRC policy on Data Sharing, there will have to be a good reason for turning down a request. Patient Information Sheets and consent forms specifically referenced making anonymised data available and this has been approved by the relevant ethics committees. Researchers given access to the data will sign data sharing agreements which will restrict the use to answering pre-specified research questions.
