# Supplementary material for: Helminth driven gut inflammation and microbial translocation associate with altered vaccine responses in rural Uganda
Source: NPJ Vaccines. 2025 Mar 26;10:56. doi: 10.1038/s41541-025-01116-x (PMC11947158; doi:10.1038/s41541-025-01116-x)
Supplement: Supplementary file 1 — Supplementary Information [file 41541_2025_1116_MOESM1_ESM.pdf]

SUPPLEMENTARY INFORMATION FOR

Helminth driven gut inflammation and microbial translocation associate with altered vaccine responses in rural Uganda

Jacent Nassuuna[1,¶], *Joas Sterk*[2,¶], *Bridgious Walusimbi*[1,3], *Agnes Natukunda*[1,4], *Ronald Nkangi*[1,5], *Rebecca Amongin*[1], *Ludoviko Zirimenya*[1,6], *Emily L Webb*[4], *Alison M Elliott*[1,6], *Gyaviira Nkurunungi*[1,3,*]

[1]Immunomodulation and Vaccines Focus Area, Vaccine Research Theme, MRC/UVRI and LSHTM Uganda Research Unit, Entebbe, Uganda

[2]University of Glasgow, Glasgow, United Kingdom.

[3]Department of Infection Biology, London School of Hygiene and Tropical Medicine, London, United Kingdom.

[4]International Statistics and Epidemiology Group, Department of Infectious Disease Epidemiology, London School of Hygiene and Tropical Medicine, London, United Kingdom.

[5]Leiden University Center for Infectious Diseases (LU-CID), Leiden University Medical Center, Leiden, The Netherlands.

[6]Department of Clinical Research, London School of Hygiene and Tropical Medicine, London, United Kingdom.

[¶]These authors contributed equally: Jacent Nassuuna, Joas Sterk.

*Email: Gyaviira.Nkurunungi@mrcuganda.org

Supplementary tables

Supplementary Table 1. Effect of baseline helminth infections on levels of markers of gut microbial translocation and gut inflammation

Marker	n	Geometric mean	cGMR (95% CI)	p	aGMR (95% CI)[‡]	p	Monte Carlo permutation p[¶]
S. mansoni							
**I-FABP2 (pg/ml)**							
CAA<30pg/ml	269	37.74	ref.		ref.		
CAA≥30pg/ml	176	39.33	1.04 (0.94, 1.16)	0.436	1.03 (0.93, 1.15)	0.531	0.998
**LBP (µg/ml)**							
CAA<30pg/ml	269	8.18	ref.		ref.		
CAA≥30pg/ml	176	8.68	1.06 (0.93, 1.21)	0.380	1.05 (0.92, 1.20)	0.494	0.997
**sCD14 (µg/ml)**							
CAA<30pg/ml	223	2.81	ref.		ref.		
CAA≥30pg/ml	156	2.69	0.96 (0.91, 1.00)	0.063	**0.94 (0.90, 0.99)**	**0.018**	0.138
**EndoCab IgG (gmu/ml)**							
CAA<30pg/ml	139	82.69	ref.		ref.		
CAA≥30pg/ml	101	95.07	1.15 (0.97, 1.37)	0.112	1.15 (0.95, 1.39)	0.155	0.736
**EndoCab IgM (mmu/ml)**							
CAA<30pg/ml	139	83.05	ref.		ref.		
CAA≥30pg/ml	101	88.37	1.06 (0.93, 1.22)	0.362	1.06 (0.92, 1.23)	0.426	0.989
**fCAL (µg/ml)**							
CAA<30pg/ml	205	25.99	ref.		ref.		
CAA≥30pg/ml	138	42.46	**1.63 (1.27, 2.10)**	**<0.001**	**1.64 (1.26, 2.13)**	**<0.001**	**0.001**
**FOB (µg/g)**							
CAA<30pg/ml	188	4.59	ref.		ref.		
CAA≥30pg/ml	129	15.84	**3.45 (2.24, 5.30)**	**<0.001**	**3.19 (2.03, 5.01)**	**<0.001**	**<0.001**
**fLcn-2 (ng/ml)**							
CAA<30pg/ml	188	102.52	ref.		ref.		
CAA≥30pg/ml	129	119.88	1.17 (0.63, 2.16)	0.617	1.16 (0.61, 2.21)	0.656	1.000
N. americanus							
**I-FABP2 (pg/ml)**							
Uninfected	336	37.23	ref.		ref.		
Infected	106	42.09	**1.131(1.00, 1.27)**	**0.043**	**1.12 (1.00, 1.26)**	**0.059**	0.397
**LBP (µg/ml)**							
Uninfected	336	8.32	ref.		ref.		
Infected	106	8.88	1.07 (0.92, 1.24)	0.394	1.07 (0.92, 1.25)	0.370	0.971
**sCD14 (µg/ml)**							
Uninfected	289	2.76	ref.		ref.		
Infected	87	2.78	1.01 (0.95, 1.08)	0.762	1.01 (0.95, 1.08)	0.767	1.000
**EndoCab IgG (gmu/ml)**							
Uninfected	179	89.26	ref.		ref.		
Infected	59	83.53	0.94 (0.77, 1.14)	0.513	0.94 (0.77, 1.15)	0.531	0.996
**EndoCab IgM (mmu/ml)**							

Uninfected	179	85.47	ref.		ref.		
Infected	59	85.92	1.01 (0.86, 1.17)	0.947	1.03 (0.89, 1.20)	0.676	1.000
**fCAL (µg/ml)**							
Uninfected	271	31.38	ref.		ref.		
Infected	72	33.08	1.05 (0.78, 1.44)	0.736	1.06 (0.78, 1.44)	0.719	1.000
**FOB (µg/g)**							
Uninfected	251	7.94	ref.		ref.		
Infected	67	6.06	0.76 (0.45, 1.31)	0.325	0.77 (0.45, 1.32)	0.335	0.952
**fLcn-2 (ng/ml)**							
Uninfected	251	114.94	ref.		ref.		
Infected	67	92.03	0.80 (0.38, 1.68)	0.555	0.81 (0.39, 1.71)	0.582	0.999
S. stercoralis							
**I-FABP2 (pg/ml)**							
Uninfected	407	38.06	ref.		ref.		
Infected	35	41.78	1.10 (0.91, 1.33)	0.332	1.09 (0.90, 1.32)	0.366	0.970
**LBP (µg/ml)**							
Uninfected	407	8.51	ref.		ref.		
Infected	35	7.75	0.91 (0.72, 1.16)	0.442	0.91 (0.72, 1.15)	0.430	0.986
**sCD14 (µg/ml)**							
Uninfected	348	2.78	ref.		ref.		
Infected	28	2.55	0.92 (0.84, 1.00)	0.061	0.92 (0.84, 1.01)	0.063	0.391
**EndoCab IgG (gmu/ml)**							
Uninfected	220	87.54	ref.		ref.		
Infected	18	91.13	1.04 (0.75, 1.44)	0.808	1.04 (0.75, 1.45)	0.802	1.000
**EndoCab IgM (mmu/ml)**							
Uninfected	220	84.89	ref.		ref.		
Infected	18	94.59	1.11 (0.87, 1.43)	0.397	1.14 (0.90, 1.46)	0.283	0.928
**fCAL (µg/ml)**							
Uninfected	314	32.08	ref.		ref.		
Infected	29	28.21	0.88 (0.56, 1.38)	0.576	0.91 (0.58, 1.42)	0.670	1.000
**FOB (µg/g)**							
Uninfected	291	7.83	ref.		ref.		
Infected	27	4.75	0.61 (0.28, 1.34)	0.214	0.61 (0.28, 1.35)	0.221	0.851
**fLcn-2 (ng/ml)**							
Uninfected	291	112.41	ref		ref.		
Infected	27	84.12	0.75 (0.25, 2.21)	0.599	0.78 (0.26, 2.30)	0.645	1.000
**Any helminth infection**†							
**I-FABP2 (pg/ml)**							
Uninfected	189	36.34	ref.		ref.		
Infected	254	39.88	1.10 (0.99, 1.22)	0.076	1.09 (0.98, 1.20)	0.114	0.633
**LBP (µg/ml)**							
Uninfected	189	8.02	ref.		ref.		
Infected	254	8.74	1.09 (0.96, 1.24)	0.195	1.09 (0.96, 1.24)	0.189	0.831
**sCD14 (µg/ml)**							
Uninfected	161	2.81	ref.		ref.		
Infected	216	2.72	0.97 (0.92, 1.02)	0.182	0.97 (0.92, 1.01)	0.164	0.781

**EndoCab IgG (gmu/ml)**							
Uninfected	99	83.24	ref.		ref.		
Infected	140	91.16	1.10 (0.92, 1.30)	0.304	1.10 (0.92, 1.31)	0.281	0.937
**EndoCab IgM (mmu/ml)**							
Uninfected	99	79.48	ref.		ref.		
Infected	140	90.03	1.13 (0.99, 1.29)	0.067	**1.16 (1.02, 1.33)**	**0.024**	0.170
**fCAL (µg/ml)**							
Uninfected	146	25.50	ref.		ref.		
Infected	196	37.35	**1.47 (1.14, 1.88)**	**0.003**	**1.49 (1.16, 1.92)**	**0.002**	**0.024**
**FOB (µg/g)**							
Uninfected	134	4.92			ref.		
Infected	183	10.45	**2.12 (1.37, 3.30)**	**0.001**	**2.16 (1.38, 3.36)**	**0.001**	**0.006**
**fLcn-2 (ng/ml)**							
Uninfected	134	111.75	ref.		ref.		
Infected	183	106.40	0.95 (0.52, 1.76)	0.875	0.97 (0.52, 1.79)	0.914	1.000

Significant differences between helminth infected and uninfected participants ($p \leq 0.05$) are highlighted in bold.
ref: Reference category
†Infection with any of *S. mansoni,* hookworm or *S. stercoralis*
‡Adjusted for age and sex, and for *S. mansoni* trial intervention arm (intensive vs standard praziquantel treatment).
¶Adjusted linear regression analyses were additionally conducted within the framework of a Monte Carlo simulation algorithm based on 1000 permutations, to adjust for multiple testing.

Abbreviations: cGMR, crude (unadjusted) geometric mean ratio; aGMR, adjusted geometric mean ratio; 95% CI, 95% confidence interval; CAA, circulating anodic antigen; I-FABP2, intestinal fatty acid-binding protein; LBP, lipopolysaccharide (LPS) binding protein; sCD14, soluble CD14; EndoCab, anti-endotoxin core antibody; fCAL, faecal calprotectin; FOB, faecal occult blood; fLcn-2, faecal lipocalin 2

23

Supplementary Table 2. Effect of baseline mono-helminth infections on levels of markers of gut microbial translocation and gut inflammation

Marker	n	Geometric mean	cGMR (95% CI)	p	aGMR (95% CI)‡	p	Monte Carlo permutation p¶
S. mansoni							
**I-FABP2 (pg/ml)**							
S. mansoni CAA<30pg/ml	189	36.34	Ref		ref.		
S. mansoni CAA≥30pg/ml	126	37.40	1.03 (0.91, 1.17)	0.65	1.03 (0.91,1.17)	0.637	1.000
**LBP (µg/ml)**							
S. mansoni CAA<30pg/ml	189	8.02	Ref		ref.		
S. mansoni CAA≥30pg/ml	126	8.53	1.06(0.91,1.24)	0.44	1.05 (0.89, 1.23)	0.590	1.000
**sCD14 (µg/ml)**							
S. mansoni CAA<30pg/ml	161	2.81	Ref		ref.		
S. mansoni CAA≥30pg/ml	112	2.72	0.97(0.91,1.02)	0.23	0.95 (0.90,1.01)	0.088	0.516
**EndoCab IgG (gmu/ml)**							
S. mansoni CAA<30pg/ml	99	83.24	Ref		ref.		
S. mansoni CAA≥30pg/ml	70	99.34	1.19(0.96, 1.49)	0.11	1.19 (0.93, 1.52)	0.166	0.761
**EndoCab IgM (mmu/ml)**							
S. mansoni CAA<30pg/ml	99	79.48	Ref		ref.		
S. mansoni CAA≥30pg/ml	70	94.32	**1.19(1.02,1.39)**	**0.03**	**1.20 (1.01, 1.42)**	**0.036**	0.264
**fCAL (µg/ml)**							
S. mansoni CAA<30pg/ml	146	25.50	Ref		ref.		
S. mansoni CAA≥30pg/ml	107	41.71	**1.64 (1.21, 2.22)**	**<0.001**	**1.53 (1.12, 2.09)**	**<0.001**	0.062
**FOB (µg/g)**							
S. mansoni CAA<30pg/ml	134	4.92	Ref		ref.		
S. mansoni CAA≥30pg/ml	100	17.44	**3.54 (2.08, 6.03)**	**<0.001**	**3.16 (1.80, 5.55)**	**<0.001**	**<0.001**
**fLcn-2(ng/ml)**							
S. mansoni CAA<30pg/ml	134	111.75	Ref		ref.		
S. mansoni CAA≥30pg/ml	100	126.37	1.13 (0.55, 2.31)	0.73	1.09 (0.51, 2.33)	0.815	1.000
N. americanus							
**I-FABP2 (pg/ml)**							
N. americanus uninfected	94	37.32	Ref		ref.		
N. americanus infected	29	44.59	1.20 (0.96, 1.49)	0.12	1.19 (0.95, 1.49)	0.128	0.624
**LBP (µg/ml)**							
N. americanus uninfected	94	8.43	Ref		ref.		
N. americanus infected	29	9.23	1.10 (0.83, 1.45)	0.53	1.10 (0.83, 1.46)	0.516	0.993
**sCD14 (µg/ml)**							
N. americanus uninfected	78	2.80	Ref		ref.		
N. americanus infected	21	2.92	1.04 (0.91, 1.20)	0.53	1.04 (0.90, 1.19)	0.630	1.000
**EndoCab IgG (gmu/ml)**							
N. americanus uninfected	51	84.14	Ref		ref.		
N. americanus infected	14	87.93	1.05 (0.73, 1.50)	0.81	1.05 (0.73, 1.50)	0.791	1.000
**EndoCab IgM (mmu/ml)**							
N. americanus uninfected	51	76.56	Ref		ref.		
N. americanus infected	14	91.37	1.19 (0.90, 1.59)	0.22	1.22 (0.91, 1.63)	0.189	0.795
**fCAL (µg/ml)**							

N. americanus uninfected	72	25.11	Ref		ref.		
N. americanus infected	19	32.33	1.29 (0.69, 2.40)	0.42	1.32 (0.69, 2.53)	0.402	0.971
**FOB (µg/g)**							
N. americanus uninfected	65	4.57	Ref		ref.		
N. americanus infected	16	4.26	0.93 (0.43, 2.01)	0.86	1.09 (0.51, 2.30)	0.826	1.000
**fLcn-2(ng/ml)**							
N. americanus uninfected	65	165.32	Ref		ref.		
N. americanus infected	16	103.16	0.62 (0.13, 2.92)	0.54	0.57(0.12, 2.84)	0.492	0.992
S. stercoralis							
**I-FABP2 (pg/ml)**							
S. stercoralis uninfected	94	37.32	Ref		ref.		
S. stercoralis infected	4	66.07	**1.77 (1.04, 3.02)**	**0.04**	1.67 (0.98, 2.86)	0.060	0.347
**LBP (µg/ml)**							
S. stercoralis uninfected	94	8.43	Ref		ref.		
S. stercoralis infected	4	7.38	0.88 (0.44,1.74)	0.70	0.83 (0.42,1.65)	0.599	0.959
**sCD14 (µg/ml)**							
S. stercoralis uninfected	78	2.80	Ref		ref.		
S. stercoralis infected	1	2.34	0.84 (0.48,1.47)	0.53	0.80 (0.46, 1.42)	0.445	0.931
**EndoCab IgG (gmu/ml)**							
S. stercoralis uninfected	51	84.14	Ref		ref.		
S. stercoralis infected	1	76.00	0.90 (0.26, 3.11)	0.87	0.89 (0.26, 3.07)	0.854	0.965
**EndoCab IgM (mmu/ml**)							
S. stercoralis uninfected	51	76.56	Ref		ref.		
S. stercoralis infected	1	181.34	2.37 (0.90, 6.23)	0.08	2.51(0.93, 6.78)	0.069	0.382
**fCAL (µg/ml)**							
S. stercoralis uninfected	72	25.11	Ref		ref.		
S. stercoralis infected	2	5.56	0.22 (0.04, 1.35)	0.10	0.21 (0.03, 1.38)	0.103	0.491
**FOB (µg/g)**							
S. stercoralis uninfected	65	4.57	Ref		ref.		
S. stercoralis infected	2	2.34	0.51(0.07, 3.79)	0.51	0.69 (0.10, 4.91)	0.708	0.965
**fLcn-2(ng/ml)**							
S. stercoralis uninfected	65	165.32	Ref		ref.		
S. stercoralis infected	2	31.91	0.19 (0.00, 11.20)	0.42	0.13 (0.00, 8.99)	0.342	0.880

Significant differences between helminth infected and uninfected participants ($p \leq 0.05$) are highlighted in bold.
ref: Reference category
‡Adjusted for age and sex, and for *S. mansoni,* trial intervention arm (intensive vs standard praziquantel treatment).
¶Adjusted linear regression analyses were additionally conducted within the framework of a Monte Carlo simulation algorithm based on 1000 permutations, to adjust for multiple testing.

Abbreviations: cGMR, crude (unadjusted) geometric mean ratio; aGMR, adjusted geometric mean ratio; 95% CI, 95% confidence interval; I-FABP2, intestinal fatty acid-binding protein; LBP, lipopolysaccharide (LPS) binding protein; sCD14, soluble CD14; EndoCab, anti-endotoxin core antibody; fCAL, faecal calprotectin; FOB, faecal occult blood; fLcn-2, faecal lipocalin 2

25

Supplementary Table 3a. Tertiles for concentrations of *S. mansoni* CAA among infected (CAA ≥30 pg/ml) participants

	**Lowest tertile**	**Middle tertile**	**Highest tertile**
CAA concentrations (range, pg/ml)	30 - 199	200 - 1960	1961 - 1726887

Supplementary Table 3b. Associations between *S. mansoni* infection intensity and levels of markers of gut microbial translocation and gut inflammation

**Marker**	**n**	**Geometric mean**	**cGMR (95% CI)**	**p**	**aGMR (95% CI)[‡]**	**p**
**I-FABP2 (pg/ml)**						
Uninfected (CAA<30pg/ml)	270	37.76	Ref		Ref	
Infected (CAA>30pg/ml): lowest tertile	50	37.68	0.95 (0.82, 1.09)	0.434	1.01 (0.85, 1.19)	0.932
Infected (CAA>30pg/ml): middle tertile	64	37.65	0.98 (0.85, 1.12)	0.760	1.00 (0.86, 1.16)	0.965
Infected (CAA>30pg/ml): highest tertile	61	42.57	1.07 (0.92, 1.23)	0.388	1.08 (0.91, 1.29)	0.385
**LBP (µg/ml)**						
Uninfected (CAA<30pg/ml)	270	8.20	Ref		Ref	
Infected (CAA>30pg/ml): lowest tertile	50	8.20	1.00 (0.84, 1.19)	0.999	1.01 (0.82, 1.25)	0.903
Infected (CAA>30pg/ml): middle tertile	64	8.74	1.04 (0.87, 1.23)	0.680	1.05 (0.87, 1.27)	0.621
Infected (CAA>30pg/ml): highest tertile	61	8.94	1.05 (0.88, 1.24)	0.600	1.03 (0.84, 1.26)	0.805
**EndoCab IgG (gmu/ml)**						
Uninfected (CAA<30pg/ml)	140	82.90	Ref		Ref	
Infected (CAA>30pg/ml): lowest tertile	22	73.09	0.88 (0.66, 1.18)	0.397	0.87 (0.65, 1.17)	0.363
Infected (CAA>30pg/ml): middle tertile	38	95.21	1.15 (0.91, 1.46)	0.254	1.16 (0.90, 1.49)	0.257
Infected (CAA>30pg/ml): highest tertile	40	109.10	**1.32 (1.05, 1.65)**	**0.018**	**1.32 (1.00, 1.76)**	**0.050**
**EndoCab IgM (gmu/ml)**						
Uninfected (CAA<30pg/ml)	140	83.04	Ref		Ref	
Infected (CAA>30pg/ml): lowest tertile	22	76.32	0.92 (0.73, 1.16)	0.469	0.93 (0.75, 1.17)	0.541
Infected (CAA>30pg/ml): middle tertile	38	87.29	1.05 (0.90, 1.23)	0.540	1.09 (0.92, 1.28)	0.314
Infected (CAA>30pg/ml): highest tertile	40	97.11	1.17 (0.99, 1.39)	0.0739	1.22 (0.99, 1.50)	0.066
**sCD14 (µg/ml)**						
Uninfected (CAA<30pg/ml)	224	2.81	Ref		Ref	
Infected (CAA>30pg/ml): lowest tertile	40	2.69	0.94 (0.87, 1.01)	0.088	0.96 (0.88, 1.04)	0.277
Infected (CAA>30pg/ml): middle tertile	57	2.72	0.95 (0.89, 1.02)	0.135	0.96 (0.89, 1.03)	0.236
Infected (CAA>30pg/ml): highest tertile	58	2.65	**0.94 (0.88, 1.00)**	**0.046**	**0.91 (0.84, 0.98)**	**0.012**
**fCAL (µg/ml)**						
Uninfected (CAA<30pg/ml)	206	26.32	Ref		Ref	
Infected (CAA>30pg/ml): lowest tertile	33	41.52	**1.73 (1.20, 2.48)**	**0.003**	**1.65 (1.06, 2.57)**	**0.026**
Infected (CAA>30pg/ml): middle tertile	52	36.38	1.24 (0.89, 1.72)	0.207	**1.43 (1.00, 2.03)**	**0.048**
Infected (CAA>30pg/ml): highest tertile	52	48.30	**1.78 (1.30, 2.45)**	**<0.001**	**1.94 (1.32, 2.84)**	**0.001**
**FOB (µg/g)**						
Uninfected (CAA<30pg/ml)	189	4.65	Ref		Ref	

Infected (CAA>30pg/ml): lowest tertile	29	7.16	1.54 (0.86, 2.77)	0.147	1.54 (0.86, 2.78)	0.149
Infected (CAA>30pg/ml): middle tertile	52	7.15	1.54 (0.96, 2.48)	0.076	**1.62 (1.00, 2.63)**	**0.051**
Infected (CAA>30pg/ml): highest tertile	47	60.91	**13.10 (7.37, 23.27)**	**<0.001**	**13.36 (6.96, 25.65)**	**<0.001**
**fLcn-2(ng/ml)**						
Uninfected (CAA<30pg/ml)	189	103.33	Ref		Ref	
Infected (CAA>30pg/ml): lowest tertile	29	112.26	1.09 (0.36, 3.28)	0.883	1.10 (0.36, 3.34)	0.868
Infected (CAA>30pg/ml): middle tertile	52	127.83	1.24 (0.52, 2.95)	0.630	1.22 (0.50, 2.95)	0.664
Infected (CAA>30pg/ml): highest tertile	47	113.02	1.09 (0.44, 2.70)	0.845	0.96 (0.35, 2.66)	0.935

Significant differences between helminth-infected and uninfected participants (p ≤ 0.05) are highlighted in bold.
ref: Reference category
‡Adjusted for age, sex and trial intervention arm (intensive vs standard praziquantel treatment).
Abbreviations: cGMR, crude (unadjusted) geometric mean ratio; aGMR, adjusted geometric mean ratio; 95% CI, 95% confidence interval; CAA, circulating anodic antigen; I-FABP2, intestinal fatty acid-binding protein; LBP, lipopolysaccharide (LPS) binding protein; sCD14, soluble CD14; EndoCab, anti-endotoxin core antibody; fCAL, faecal calprotectin; FOB, faecal occult blood; fLcn-2, faecal lipocalin 2

Supplementary Table 4a. Impact of intensive vs standard praziquantel treatment on levels of markers of gut microbial translocation and gut inflammation in participants who were *Sm* infected (CAA ≥30 pg/ml) at baseline (screening) but were *Sm* uninfected (CAA <30 pg/ml) by week 0 in the intensive arm.

Marker	n	Geometric mean	aGMR (95% CI)‡	p	Monte Carlo permutation p¶
**I-FABP2 (pg/ml)**					
Standard	103	40.32	ref.		
Intensive	64	39.02	0.97 (0.83, 1.14)	0.726	1.000
**LBP (µg/ml)**					
Standard	103	8.62	ref.		
Intensive	64	7.94	0.92 (0.74, 1.15)	0.463	0.996
**sCD14 (µg/ml)**					
Standard	94	2.77	ref.		
Intensive	57	2.75	0.99 (0.94, 1.06)	0.846	1.000
**EndoCab IgG (gmu/ml)**					
Standard	64	94.89	ref.		
Intensive	38	83.73	0.88 (0.67, 1.17)	0.380	0.980
**EndoCab IgM (mmu/ml)**					
Standard	64	94.11	ref.		
Intensive	38	82.91	0.90 (0.72, 1.11)	0.313	0.960
**fCAL(µg/ml)**					
Standard	85	47.06	ref.		
Intensive	47	25.09	**0.57 (0.39, 0.81)**	**0.002**	**0.018**
**FOB (µg/g)**					
Standard	79	21.22	ref.		
Intensive	46	5.48	**0.27 (0.12, 0.63)**	**0.003**	**0.019**
**fLcn-2 (ng/ml)**					
Standard	79	108.34	ref.		
Intensive	46	52.26	0.51 (0.19, 1.35)	0.174	0.793

ref: Reference category
‡Adjusted for age and sex
¶Linear regression analyses adjusting for age and sex were additionally conducted within the framework of a Monte Carlo simulation algorithm based on 1000 permutations, to adjust for multiple testing.

Abbreviations: aGMR, adjusted geometric mean ratio; 95% CI, 95% confidence interval; I-FABP2, intestinal fatty acid-binding protein; LBP, lipopolysaccharide (LPS) binding protein; sCD14, soluble CD14; EndoCab, anti-endotoxin core antibody; fCAL, faecal calprotectin; FOB, faecal occult blood; fLcn-2, faecal lipocalin 2

Supplementary Table 4b. Impact of intensive vs standard praziquantel treatment on levels of markers of gut microbial translocation and gut inflammation among rural (POPVAC A) participants – irrespective of baseline *S. mansoni* infection status

Marker	n	Geometric mean	GMR (95% CI)	p	Monte Carlo permutation p¶
**I-FABP2 (pg/ml)**					
Standard	218	39.18	ref.		
Intensive	227	37.60	0.96 (0.87, 1.06)	0.425	0.981
**LBP (µg/ml)**					
Standard	218	8.76	ref.		
Intensive	227	8.02	0.92 (0.80, 1.04)	0.178	0.785
**sCD14 (µg/ml)**					
Standard	175	2.82	ref.		
Intensive	204	2.71	0.96 (0.92, 1.01)	0.116	0.627
**EndoCab IgG (gmu/ml)**					
Standard	101	91.04	ref.		
Intensive	139	85.33	0.94 (0.79, 1.11)	0.462	0.990
**EndoCab IgM (mmu/ml)**					
Standard	101	88.65	ref.		
Intensive	139	82.86	0.94 (0.82, 1.07)	0.321	0.933
**fCAL(µg/ml)**					
Standard	170	35.37	ref.		
Intensive	176	28.38	0.80 (0.63, 1.03)	0.082	0.489
**FOB (µg/g)**					
Standard	152	10.31	ref.		
Intensive	168	5.70	**0.55 (0.36, 0.85)**	**0.008**	**0.063**
**fLcn-2 (ng/ml)**					
Standard	152	119.45	ref.		
Intensive	168	101.50	0.85 (0.47, 1.55)	0.594	0.999

ref: Reference category
¶Linear regression analyses were additionally conducted within the framework of a Monte Carlo simulation algorithm based on 1000 permutations, to adjust for multiple testing.

Abbreviations: GMR, geometric mean ratio; 95% CI, 95% confidence interval; I-FABP2, intestinal fatty acid-binding protein; LBP, lipopolysaccharide (LPS) binding protein; sCD14, soluble CD14; EndoCab, anti-endotoxin core antibody; fCAL, faecal calprotectin; FOB, faecal occult blood; fLcn-2, faecal lipocalin 2

Supplementary Table 5. Associations between levels of markers of gut microbial translocation / gut inflammation and vaccine-specific responses measured at the POPVAC primary endpoint (week 8, or, for Td, at week 52)

Marker	Crude β (95% CI)	p	Adjusted β (95% CI)‡	p	Monte Carlo permutation p¶
**BCG-specific IFN-γ response, 8 weeks post-vaccination**					
I-FABP2	0.05 (-0.11, 0.21)	0.548	0.06 (-0.10, 0.22)	0.477	0.993
LBP	**-0.16 (-0.28, -0.03)**	**0.014**	**-0.15 (-0.28, -0.03)**	**0.017**	0.118
sCD14	**0.43 (0.04, 0.82)**	**0.030**	**0.44 (0.05, 0.83)**	**0.026**	0.178
EndoCab IgG	0.02 (-0.15, 0.19)	0.826	0.01 (-0.16, 0.18)	0.893	1.000
EndoCab IgM	-0.18 (-0.41, 0.04)	0.109	-0.16 (-0.39, 0.07)	0.182	0.796
fCAL	**-0.12 (-0.20, -0.05)**	**0.002**	**-0.12 (-0.20, -0.04)**	**0.003**	**0.025**
FOB	**-0.06 (-0.10, -0.01)**	**0.017**	**-0.06 (-0.10, -0.01)**	**0.015**	0.112
fLcn-2	**-0.06 (-0.09, -0.03)**	**0.001**	**-0.06 (-0.09, -0.02)**	**0.001**	**0.006**
**Yellow Fever PRNT50 titres, 4 weeks post-vaccination**					
I-FABP2	-0.07 (-0.32, 0.18)	0.594	-0.08 (-0.33, 0.17)	0.543	0.999
LBP	-0.13 (-0.32, 0.07)	0.197	-0.14 (-0.33, 0.06)	0.170	0.790
sCD14	-0.31 (-0.98, 0.35)	0.358	-0.33 (-1.00, 0.34)	0.331	0.964
EndoCab IgG	-0.08 (-0.34, 0.18)	0.551	-0.07 (-0.34, 0.19)	0.583	1.000
EndoCab IgM	0.19 (-0.15, 0.53)	0.273	0.19 (-0.16, 0.54)	0.291	0.942
fCAL	-0.12 (-0.25, 0.002)	0.054	**-0.13 (-0.26, -0.005)**	**0.042**	0.293
FOB	0.0003 (-0.08, 0.08)	0.993	0.0003 (-0.08, 0.08)	0.993	1.000
fLcn-2	0.01 (-0.05, 0.06)	0.774	0.01 (-0.05, 0.06)	0.795	1.000
**Yellow Fever PRNT90 titres, 4 weeks post-vaccination**					
I-FABP2	-0.06 (-0.29, 0.18)	0.634	-0.08 (-0.32, 0.16)	0.532	0.998
LBP	-0.14 (-0.33, 0.04)	0.124	-0.16 (-0.34, 0.03)	0.098	0.574
sCD14	-0.23 (-0.86, 0.41)	0.482	-0.26 (-0.89, 0.38)	0.426	0.986
EndoCab IgG	-0.11 (-0.37, 0.14)	0.387	-0.10 (-0.36, 0.15)	0.434	0.989
EndoCab IgM	0.24 (-0.09, 0.57)	0.150	0.25 (-0.09, 0.58)	0.149	0.743
fCAL	**-0.14 (-0.25, -0.02)**	**0.025**	**-0.15 (-0.27, -0.03)**	**0.016**	0.127
FOB	0.01 (-0.07, 0.08)	0.890	0.01 (-0.07, 0.08)	0.888	1.000
fLcn-2	0.01 (-0.04, 0.07)	0.676	0.01 (-0.04, 0.06)	0.726	1.000
***Salmonella* Typhi O:LPS-specific IgG, 4 weeks post-vaccination**					
I-FABP2	-0.18 (-0.39, 0.04)	0.106	-0.18 (-0.40, 0.04)	0.107	0.607
LBP	0.07 (-0.10, 0.23)	0.439	0.07 (-0.10, 0.24)	0.434	0.987
sCD14	-0.05 (-0.60, 0.49)	0.850	-0.06 (-0.61, 0.49)	0.840	1.000
EndoCab IgG	**0.43 (0.21, 0.65)**	**<0.001**	**0.43 (0.21, 0.65)**	**<0.001**	**0.002**
EndoCab IgM	0.15 (-0.15, 0.45)	0.325	0.19 (-0.12, 0.49)	0.225	0.875
fCAL	0.09 (-0.02, 0.19)	0.101	0.09 (-0.01, 0.20)	0.087	0.528
FOB	0.01 (-0.05, 0.08)	0.697	0.12 (-0.05, 0.08)	0.701	1.000
fLcn-2	-0.003 (-0.05, 0.04)	0.890	-0.002 (-0.05, 0.05)	0.941	1.000
**HPV-16-specific IgG, 4 weeks post-vaccination**					
I-FABP2	**-0.26 (-0.47, -0.04)**	**0.019**	**-0.24 (-0.44, -0.03)**	**0.025**	0.171
LBP	-0.01 (-0.18, 0.16)	0.930	-0.04 (-0.20, 0.12)	0.652	1.000
sCD14	-0.26 (-0.82, 0.31)	0.371	-0.26 (-0.80, 0.27)	0.337	0.960
EndoCab IgG	0.06 (-0.19, 0.31)	0.632	0.06 (-0.17, 0.30)	0.598	1.000
EndoCab IgM	0.17 (-0.15, 0.49)	0.295	-0.03 (-0.34, 0.28)	0.853	1.000
fCAL	-0.05 (-0.06, 0.16)	0.336	0.03 (-0.07, 0.14)	0.531	0.995
FOB	0.03 (-0.03, 0.10)	0.327	0.03 (-0.03, 0.09)	0.301	0.939
fLcn-2	0.03 (-0.01, 0.08)	0.164	0.03 (-0.02, 0.07)	0.281	0.933
**HPV-18-specific IgG, 4 weeks post-vaccination**					
I-FABP2	**-0.26 (-0.43, -0.08)**	**0.004**	**-0.25 (-0.42, -0.07)**	**0.005**	**0.041**
LBP	-0.07 (-0.21, 0.07)	0.331	-0.09 (-0.22, 0.05)	0.200	0.839
sCD14	-0.15 (-0.60, 0.31)	0.527	-0.15 (-0.60, 0.29)	0.498	0.997
EndoCab IgG	0.14 (-0.06, 0.34)	0.166	0.14 (-0.05, 0.33)	0.154	0.742
EndoCab IgM	0.17 (-0.08, 0.43)	0.179	0.05 (-0.20, 0.30)	0.687	1.000
fCAL	-0.02 (-0.11, 0.07)	0.700	-0.03 (-0.12, 0.06)	0.483	0.997
FOB	0.03 (-0.20, 0.09)	0.220	0.03 (-0.02, 0.09)	0.208	0.853
fLcn-2	0.01 (-0.03, 0.06)	0.467	0.01 (-0.03, 0.05)	0.639	1.000
**Tetanus toxoid-specific IgG, 24 weeks post-vaccination**					
I-FABP2	0.08 (-0.12, 0.29)	0.432	0.09 (-0.12, 0.29)	0.398	0.983
LBP	-0.08 (-0.24, 0.07)	0.278	-0.07 (-0.22, 0.08)	0.352	0.958
sCD14	-0.47 (-0.95, 0.02)	0.058	**-0.47 (-0.95, 0.002)**	**0.051**	0.329
EndoCab IgG	-0.11 (-0.31, 0.10)	0.296	-0.12 (-0.32, 0.08)	0.236	0.881
EndoCab IgM	-0.26 (-0.53, 0.01)	0.063	-0.19 (-0.48, 0.10)	0.192	0.808
fCAL	-0.08 (-0.18, 0.02)	0.107	-0.06 (-0.16, 0.04)	0.256	0.902
FOB	-0.03 (-0.10, 0.03)	0.327	-0.03 (-0.09, 0.03)	0.351	0.957
fLcn-2	-0.0003 (-0.05, 0.05)	0.989	0.005 (-0.04, 0.05)	0.842	1.000
**Diphtheria toxoid-specific IgG, 24 weeks post-vaccination**					
I-FABP2	0.01 (-0.09, 0.11)	0.842	0.02 (-0.08, 0.13)	0.646	0.999
LBP	-0.03 (-0.10, 0.05)	0.509	-0.02 (-0.10, 0.06)	0.571	0.997
sCD14	**-0.28 (-0.52, -0.04)**	**0.024**	**-0.27 (-0.51, -0.03)**	**0.030**	0.226
EndoCab IgG	0.05 (-0.05, 0.15)	0.307	0.04 (-0.06, 0.14)	0.399	0.980

EndoCab IgM	0.07 (-0.07, 0.20)	0.341	0.09 (-0.06, 0.23)	0.237	0.886
fCAL	-0.02 (-0.06, 0.03)	0.517	-0.12 (-0.16, 0.04)	0.615	0.997
FOB	-0.01 (-0.04, 0.02)	0.693	-0.01 (-0.04, 0.02)	0.715	1.000
fLcn-2	-0.01 (-0.03, 0.01)	0.525	-0.01 (-0.03, 0.02)	0.551	0.997

‡Adjusted for age and sex

¶Adjusted linear regression analyses were additionally conducted within the framework of a Monte Carlo simulation algorithm based on 1000 permutations, to adjust for multiple testing.

Abbreviations: β, regression coefficient; 95% CI, 95% confidence interval; IFN-ɣ, interferon gamma; BCG, Bacillus Calmette Guérin; PRNT, plaque reduction neutralisation test; HPV, human papillomavirus I-FABP2, intestinal fatty acid-binding protein; LBP, lipopolysaccharide (LPS) binding protein; sCD14, soluble CD14; EndoCab, anti-endotoxin core antibodies; fCAL, faecal calprotectin; FOB, faecal occult blood; fLcn-2, faecal lipocalin-2

Supplementary Table 6. Associations between levels of markers of gut microbial translocation / gut inflammation and vaccine-specific responses measured at the POPVAC secondary endpoint (week 52)

Marker	Crude β (95% CI)	p	Adjusted β (95% CI)‡	p	Monte Carlo permutation p¶
**BCG-specific IFN-γ response, 52 weeks post-vaccination**					
I-FABP2	-0.06 (-0.23, 0.12)	0.525	-0.04 (-0.21, 0.14)	0.667	1.000
LBP	-0.09 (-0.22, 0.04)	0.162	-0.08 (-0.21, 0.05)	0.207	0.825
sCD14	-0.02 (-0.45, 0.41)	0.928	-0.01 (-0.44, 0.42)	0.950	1.000
EndoCab IgG	0.05 (-0.15, 0.25)	0.612	0.04 (-0.15, 0.24)	0.668	1.000
EndoCab IgM	0.03 (-0.23, 0.29)	0.810	0.14 (-0.14, 0.42)	0.313	0.946
fCAL	-0.06 (-0.15, 0.03)	0.187	-0.05 (-0.14, 0.04)	0.280	0.923
FOB	-0.03 (-0.09, 0.03)	0.306	-0.03 (-0.08, 0.03)	0.353	0.965
fLcn-2	-0.02 (-0.06, 0.02)	0.330	-0.02 (-0.06, 0.02)	0.398	0.983
**Yellow Fever PRNT50 titres, 48 weeks post-vaccination**					
I-FABP2	-0.08 (-0.33, 0.17)	0.532	-0.02 (-0.27, 0.23)	0.896	1.000
LBP	-0.12 (-0.30, 0.07)	0.208	-0.11 (-0.30, 0.07)	0.219	0.862
sCD14	0.24 (-0.37, 0.85)	0.442	0.27 (-0.34, 0.87)	0.388	0.973
EndoCab IgG	-0.01 (-0.29, 0.27)	0.941	0.001 (-0.28, 0.28)	0.992	1.000
EndoCab IgM	0.18 (-0.19, 0.56)	0.329	0.08 (-0.31, 0.48)	0.673	1.000
fCAL	0.01 (-0.11, 0.13)	0.884	0.01 (-0.11, 0.13)	0.857	1.000
FOB	-0.02 (-0.10, 0.06)	0.662	-0.02 (-0.09, 0.06)	0.680	1.000
fLcn-2	0.04 (-0.01, 0.10)	0.124	0.04 (-0.01, 0.10)	0.118	0.640
**Yellow Fever PRNT90 titres, 48 weeks post-vaccination**					
I-FABP2	-0.21 (-0.47, 0.05)	0.117	-0.14 (-0.40, 0.13)	0.303	0.949
LBP	-0.14 (-0.34, 0.05)	0.150	-0.14 (-0.33, 0.06)	0.160	0.763
sCD14	0.32 (-0.33, 0.98)	0.334	0.35 (-0.30, 1.00)	0.290	0.939
EndoCab IgG	0.03 (-0.27, 0.33)	0.847	0.05 (-0.25, 0.34)	0.764	1.000
EndoCab IgM	0.17 (-0.23, 0.57)	0.397	0.06 (-0.37, 0.48)	0.789	1.000
fCAL	0.02 (-0.10, 0.15)	0.707	0.03 (-0.10, 0.16)	0.654	1.000
FOB	-0.003 (-0.09, 0.08)	0.944	-0.002 (-0.08, 0.08)	0.970	1.000
fLcn-2	0.05 (-0.01, 0.11)	0.086	0.05 (-0.01, 0.11)	0.078	0.487
***Salmonella* Typhi O:LPS-specific IgG, 48 weeks post-vaccination**					
I-FABP2	-0.13 (-0.37, 0.12)	0.311	-0.09 (-0.34, 0.16)	0.471	1.000
LBP	-0.01 (-0.19, 0.17)	0.909	0.001 (-0.18, 0.18)	0.991	1.000
sCD14	-0.16 (-0.75, 0.42)	0.585	-0.16 (-0.75, 0.43)	0.588	1.000
EndoCab IgG	**0.53 (0.28, 0.78)**	**<0.001**	**0.51 (0.26, 0.76)**	**<0.001**	**<0.001**
EndoCab IgM	0.22 (-0.14, 0.58)	0.228	0.25 (-0.13, 0.63)	0.194	0.812
fCAL	0.10 (-0.01, 0.22)	0.086	**0.12 (0.01, 0.24)**	**0.041**	0.245
FOB	0.03 (-0.05, 0.10)	0.450	0.03 (-0.04, 0.10)	0.431	0.993
fLcn-2	0.02 (-0.04, 0.07)	0.584	0.02 (-0.03, 0.07)	0.471	0.997
**HPV-16-specific IgG, 48 weeks post-vaccination**					
I-FABP2	-0.13 (-0.42, 0.17)	0.389	-0.03 (-0.32, 0.27)	0.853	1.000
LBP	0.09 (-0.13, 0.31)	0.407	0.09 (-0.13, 0.30)	0.421	0.989
sCD14	**-0.83 (-1.55, -0.11)**	**0.024**	**-0.79 (-1.50, -0.07)**	**0.031**	0.263
EndoCab IgG	0.24 (-0.10, 0.57)	0.164	0.23 (-0.10, 0.56)	0.175	0.815
EndoCab IgM	0.09 (-0.36, 0.54)	0.697	-0.05 (-0.52, 0.42)	0.834	1.000
fCAL	-0.02 (-0.16, 0.12)	0.755	-0.03 (-0.16, 0.11)	0.698	1.000
FOB	0.07 (-0.02, 0.16)	0.105	0.07 (-0.01, 0.16)	0.092	0.563
fLcn-2	0.02 (-0.04, 0.09)	0.451	0.02 (-0.04, 0.09)	0.478	0.995
**HPV-18-specific IgG, 48 weeks post-vaccination**					
I-FABP2	-0.04 (-0.27, 0.20)	0.765	0.02 (-0.22, 0.25)	0.891	1.000
LBP	-0.01 (-0.19, 0.16)	0.881	-0.02 (-0.20, 0.15)	0.785	1.000
sCD14	**-0.60 (-1.13, -0.06)**	**0.029**	**-0.57 (-1.11, -0.04)**	**0.035**	0.258
EndoCab IgG	0.15 (-0.10, 0.40)	0.236	0.15 (-0.10, 0.40)	0.236	0.878
EndoCab IgM	0.20 (-0.13, 0.54)	0.235	0.15 (-0.21, 0.51)	0.409	0.981
fCAL	-0.00 (-0.11, 0.10)	0.989	-0.01 (-0.12, 0.09)	0.815	1.000
FOB	0.01 (-0.05, 0.08)	0.692	0.01 (-0.05, 0.08)	0.685	1.000
fLcn-2	0.02 (-0.03, 0.06)	0.543	0.01 (-0.04, 0.06)	0.624	1.000

‡Adjusted for age and sex
¶Adjusted linear regression analyses were additionally conducted within the framework of a Monte Carlo simulation algorithm based on 1000 permutations, to adjust for multiple testing.
Abbreviations: β, regression coefficient; 95% CI, 95% confidence interval; IFN-γ, interferon gamma; BCG, Bacillus Calmette Guérin; PRNT, plaque reduction neutralisation test; HPV, human papillomavirus I-FABP2, intestinal fatty acid-binding protein; LBP, lipopolysaccharide (LPS) binding protein; sCD14, soluble CD14; EndoCab, anti-endotoxin core antibodies; fCAL, faecal calprotectin; FOB, faecal occult blood; fLcn-2, faecal lipocalin-2

Supplementary Table 7. Associations between levels of markers of gut microbial translocation / gut inflammation and the absolute increase in vaccine-specific responses from baseline (pre-vaccination) to the primary endpoint

Marker	Crude β (95% CI)	p	Adjusted β (95% CI)‡	p	Monte Carlo permutation p¶
**Absolute increase in BCG-specific IFN-γ response from week 0 (pre-vaccination) to 8 weeks post-BCG vaccination**					
I-FABP2	-0.10 (-0.56, 0.36)	0.668	-0.09 (-0.57, 0.38)	0.696	1.000
LBP	-0.24 (-0.59, 0.10)	0.169	-0.25 (-0.59, 0.10)	0.166	0.761
sCD14	1.18 (-0.05, 2.41)	0.059	1.18 (-0.06, 2.42)	0.061	0.398
EndoCab IgG	-0.22 (-0.75, 0.32)	0.423	-0.25 (-0.79, 0.30)	0.371	0.973
EndoCab IgM	-0.57 (-1.17, 0.04)	0.065	-0.58 (-1.21, 0.04)	0.067	0.436
fCAL	-0.23 (-0.46, 0.01)	0.061	-0.23 (0.47, 0.01)	0.061	0.400
FOB	-0.07 (-0.20, 0.06)	0.294	-0.07 (-0.20, 0.06)	0.296	0.943
fLcn-2	-0.10 (-0.21, 0.001)	0.053	-0.10 (-0.21, 0.003)	0.058	0.377
**Absolute increase in *Salmonella* Typhi O:LPS-specific IgG from week 0 (pre-vaccination) to 4 weeks post-Ty21a vaccination**					
I-FABP2	-0.18 (-0.53, 0.17)	0.321	-0.20 (-0.56, 0.16)	0.274	0.924
LBP	0.13 (-0.15, 0.40)	0.356	0.12 (-0.16, 0.40)	0.389	0.978
sCD14	**1.06 (0.13, 1.99)**	**0.025**	**1.03 (0.10, 1.96)**	**0.030**	0.213
EndoCab IgG	0.25 (-0.90, 0.59)	0.144	0.26 (-0.09, 0.60)	0.141	0.688
EndoCab IgM	0.13 (-0.31, 0.57)	0.561	0.20 (-0.25, 0.66)	0.382	0.976
fCAL	-0.01 (-0.18, 0.17)	0.942	-0.01 (-0.19, 0.17)	0.887	1.000
FOB	-0.04 (-0.15, 0.06)	0.431	-0.04 (-0.15, 0.06)	0.434	0.987
fLcn-2	-0.01 (-0.09, 0.07)	0.790	-0.01 (-0.09, 0.07)	0.791	1.000
**Absolute increase in HPV-16-specific IgG from week 0 (pre-vaccination) to 4 weeks post-HPV vaccination**					
I-FABP2	**-0.28 (-0.51, -0.04)**	**0.021**	**-0.26 (-0.49, -0.03)**	**0.024**	0.166
LBP	0.004 (-0.18, 0.19)	0.964	-0.03 (-0.20, 0.15)	0.760	1.000
sCD14	-0.21 (-0.83, 0.40)	0.494	-0.23 (-0.82, 0.36)	0.446	0.995
EndoCab IgG	0.01 (-0.27, 0.29)	0.960	0.01 (-0.25, 0.28)	0.925	1.000
EndoCab IgM	0.25 (-0.11, 0.60)	0.175	0.06 (-0.29, 0.41)	0.727	1.000
fCAL	0.05 (-0.06, 0.17)	0.370	0.03 (-0.08, 0.14)	0.584	1.000
FOB	0.03 (-0.04, 0.10)	0.372	0.03 (-0.04, 0.10)	0.348	0.975
fLcn-2	0.03 (-0.02, 0.08)	0.261	0.02 (-0.03, 0.07)	0.420	1.000
**Absolute increase in HPV-18-specific IgG from week 0 (pre-vaccination) to 4 weeks post-HPV vaccination**					
I-FABP2	**-0.29 (-0.51, -0.08)**	**0.008**	**-0.28 (-0.49, -0.06)**	**0.011**	0.083
LBP	-0.07 (-0.24, 0.10)	0.421	-0.09 (-0.26, 0.08)	0.291	0.939
sCD14	-0.15 (-0.71, 0.42)	0.606	-0.15 (-0.71, 0.40)	0.589	1.000
EndoCab IgG	0.12 (-0.13, 0.38)	0.327	0.12 (-0.12, 0.37)	0.323	0.958
EndoCab IgM	0.21 (-0.11, 0.53)	0.196	0.09 (-0.23, 0.42)	0.574	1.000
fCAL	-0.03 (-0.14, 0.07)	0.541	-0.05 (-0.15, 0.06)	0.373	0.977
FOB	0.04 (-0.02, 0.11)	0.209	0.04 (-0.02, 0.11)	0.199	0.810
fLcn-2	0.01 (-0.04, 0.06)	0.609	0.01 (-0.04, 0.06)	0.776	1.000
**Absolute increase in Tetanus toxoid-specific IgG from week 0 (pre-vaccination) to 24 weeks post-tetanus vaccination**					
I-FABP2	0.09 (-0.12, 0.29)	0.414	0.09 (-0.11, 0.30)	0.382	0.970
LBP	-0.09 (-0.24, 0.06)	0.248	-0.08 (-0.23, 0.07)	0.312	0.931
sCD14	-0.48 (-0.96, 0.002)	0.051	**-0.49 (-0.96, -0.01)**	**0.046**	0.311
EndoCab IgG	-0.11 (-0.31, 0.10)	0.294	-0.12 (-0.32, 0.08)	0.239	0.873
EndoCab IgM	-0.27 (-0.54, 0.004)	0.054	-0.21 (-0.50, 0.08)	0.150	0.708
fCAL	-0.08 (-0.18, 0.02)	0.130	-0.05 (-0.15, 0.05)	0.286	0.912
FOB	-0.03 (-0.09, 0.03)	0.356	-0.03 (-0.09, 0.04)	0.382	0.970
fLcn-2	0.0003 (-0.05, 0.05)	0.990	0.01 (-0.04, 0.05)	0.831	1.000
**Absolute increase in diphtheria toxoid-specific IgG from week 0 (pre-vaccination) to 24 weeks post-diphtheria vaccination**					
I-FABP2	0.01 (-0.08, 0.11)	0.772	0.02 (-0.08, 0.12)	0.676	1.000
LBP	0.01 (-0.06, 0.08)	0.789	0.01 (-0.06, 0.08)	0.772	1.000
sCD14	**-0.32 (-0.54, -0.09)**	**0.007**	**-0.31 (-0.54, -0.08)**	**0.009**	0.069
EndoCab IgG	0.03 (-0.06, 0.12)	0.554	0.02 (-0.07, 0.12)	0.620	1.000
EndoCab IgM	0.10 (-0.02, 0.22)	0.110	0.11 (-0.02, 0.24)	0.092	0.530
fCAL	0.002 (-0.04, 0.05)	0.932	0.001 (-0.05, 0.05)	0.981	1.000
FOB	-0.002 (-0.30, 0.03)	0.872	-0.002 (-0.03, 0.03)	0.872	1.000
fLcn-2	-0.002 (-0.02, 0.02)	0.884	0.001 (-0.02, 0.02)	0.915	1.000

‡Adjusted for age and sex

¶Adjusted linear regression analyses were additionally conducted within the framework of a Monte Carlo simulation algorithm based on 1000 permutations, to adjust for multiple testing.

Abbreviations: β, regression coefficient; 95% CI, 95% confidence interval; IFN-γ, interferon gamma; BCG, Bacillus Calmette Guérin; PRNT, plaque reduction neutralisation test; HPV, human papillomavirus I-FABP2, intestinal fatty acid-binding protein; LBP, lipopolysaccharide (LPS) binding protein; sCD14, soluble CD14; EndoCab, anti-endotoxin core antibodies; fCAL, faecal calprotectin; FOB, faecal occult blood; fLcn-2, faecal lipocalin-2

Supplementary Table 8. Associations between levels of markers of gut microbial translocation / gut inflammation and protective vaccine-specific responses

Marker			Odds Ratio (95% CI)	p	Adjusted Odds Ratio (95% CI)**	p
***Salmonella* Typhi O:LPS-specific IgG, 4 weeks post-vaccination**						
	number seronegative‡*	number (%) seropositive¶				
I-FABP2	21	344 (94)	0.49 (0.07, 3.45)	0.477	0.46 (0.07, 3.28)	0.441
LBP	21	344 (94)	1.26 (0.30, 5.21)	0.751	1.29 (0.31, 5.33)	0.723
sCD14	22	295 (93)	27.00 (0.68, 1079.36)	0.080	24.53 (0.62, 964.26)	0.088
EndoCab IgG	7	187 (96)	0.97 (0.08, 12.00)	0.979	0.99 (0.08, 12.04)	0.991
EndoCab IgM	7	187 (96)	0.41 (0.01, 12.22)	0.603	0.82 (0.03, 21.58)	0.906
fCAL	21	302 (93)	0.53 (0.22, 1.25)	0.146	0.52 (0.22, 1.26)	0.149
FOB	19	278 (94)	0.84 (0.52, 1.35)	0.470	0.84 (0.52, 1.35)	0.468
fLcn-2	19	278 (94)	0.91 (0.61, 1.37)	0.660	0.92 (0.61, 1.40)	0.713
**Tetanus toxoid-specific IgG, 24 weeks post-vaccination**						
	number unprotected‡*	number (%) protected¶				
I-FABP2	6	303 (98)	15.21 (0.43, 543.37)	0.136	26.30 (0.62, 1116.18)	0.087
LBP	6	303 (98)	0.86 (0.05, 13.89)	0.913	0.80 (0.05, 13.40)	0.880
sCD14	3	261 (99)	3.62 (0.00, 112429.55)	0.807	19.93 (0.00, 6.24e+06)	0.643
EndoCab IgG	2	158 (99)	0.58 (0.01, 43.79)	0.806	0.12 (0.00, 20.71)	0.422
EndoCab IgM	2	158 (99)	0.10 (0.00, 48.32)	0.463	4.33e-10 (9.22e-26, 2.03e+06	0.242
fCAL	5	244 (98)	0.37 (0.06, 2.14)	0.265	0.36 (0.06, 2.06)	0.252
FOB	4	228 (98)	3.24 (0.23, 45.89)	0.384	3.49 (0.18, 67.80)	0.410
fLcn-2	4	228 (98)	1.50 (0.68, 3.30)	0.311	1.58 (0.71, 3.48)	0.260

*Reference group
‡Number of participants with <4-fold increase in *S.* Typhi O:LPS-specific IgG from baseline or number of participants with tetanus toxoid-specific IgG levels <0.1 IU/ml
¶ Number of participants with ≥4-fold increase in *S.* Typhi O:LPS-specific IgG from baseline or Number of participants with protective tetanus toxoid-specific IgG levels (≥0.1 IU/ml)
**Adjusted for age and sex
Abbreviations: 95% CI, 95% confidence interval; I-FABP2, intestinal fatty acid-binding protein; LBP, lipopolysaccharide (LPS) binding protein; sCD14, soluble CD14; EndoCab, anti-endotoxin core antibodies; fCAL, faecal calprotectin; FOB, faecal occult blood; fLcn-2, faecal lipocalin-2

Supplementary figures

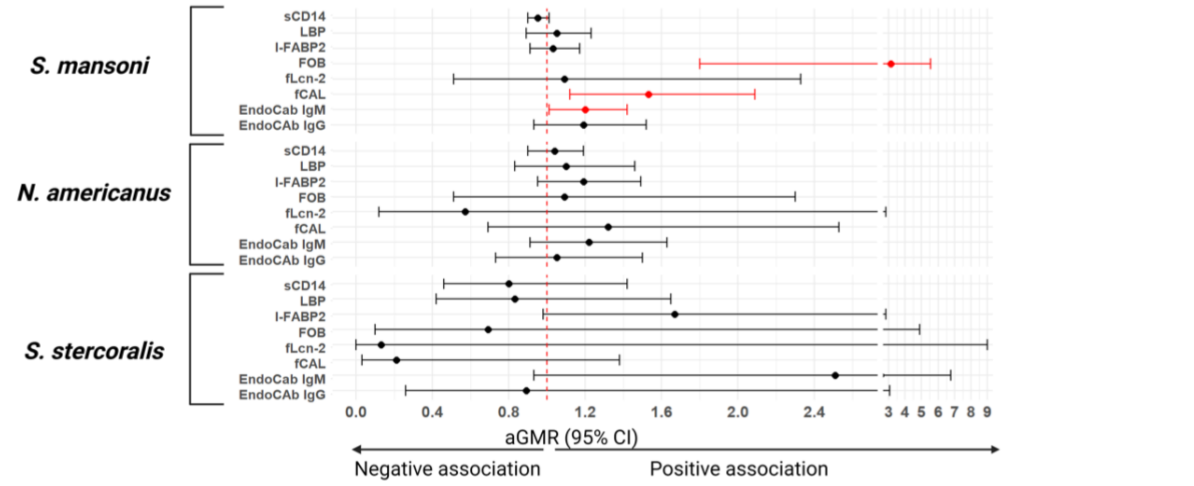

Supplementary Figure 1. Effect of mono-helminth infections on levels of markers of gut microbial translocation/gut inflammation.

Abbreviations: aGMR, adjusted geometric mean ratio; 95% CI, 95% confidence interval; I-FABP2, intestinal fatty acid-binding protein; LBP, lipopolysaccharide (LPS) binding protein; sCD14, soluble CD14; EndoCab, anti-endotoxin core antibodies; fCAL, faecal calprotectin; FOB, faecal occult blood; fLcn-2, faecal lipocalin-2

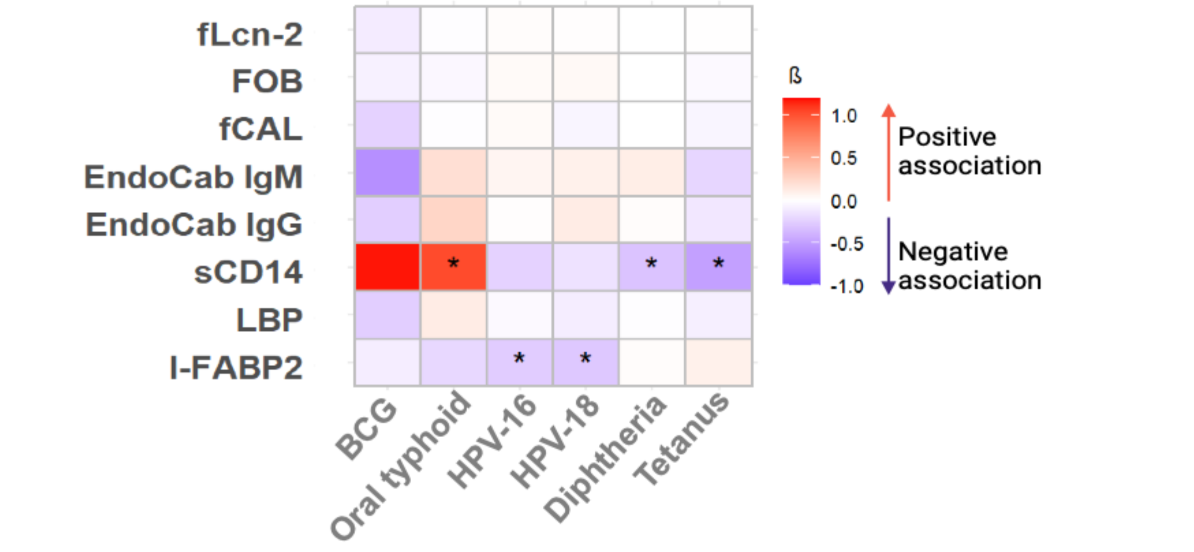

Supplementary Figure 2. Associations between levels of markers of gut microbial translocation / gut inflammation and vaccine responses (assessed as the absolute increase in vaccine-specific response from baseline [pre-vaccination] to the primary endpoint [week 8, or, for Td, at week 52]).
Analyses adjusted for age and sex. Asterisks (*) represent significant associations.

*p<0.05; **p<0.01; ***p<0.001
Abbreviations: β, regression coefficient; BCG, Bacillus Calmette Guérin; HPV, human papillomavirus; I-FABP2, intestinal fatty acid-binding protein; LBP, lipopolysaccharide (LPS) binding protein; sCD14, soluble CD14; EndoCab, anti-endotoxin core antibodies; fCAL, faecal calprotectin; FOB, faecal occult blood; fLcn-2, faecal lipocalin-2
